# Dynamic 3D genome reorganization during development and metabolic stress of the porcine liver

**DOI:** 10.1038/s41421-022-00416-z

**Published:** 2022-06-14

**Authors:** Luxi Chen, Jing Li, Renqiang Yuan, Yujie Wang, Jiaman Zhang, Yu Lin, Lina Wang, Xingxing Zhu, Wei Zhu, Jingyi Bai, Fanli Kong, Bo Zeng, Lu Lu, Jideng Ma, Keren Long, Long Jin, Zhiqing Huang, Jinlong Huo, Yiren Gu, Danyang Wang, Delin Mo, Diyan Li, Qianzi Tang, Xuewei Li, Jiangwei Wu, Yaosheng Chen, Mingzhou Li

**Affiliations:** 1 State Key Laboratory of Biocontrol, School of Life Sciences, Sun Yat-sen University, Guangzhou, Guangdong China; 2 Institute of Animal Genetics and Breeding, College of Animal Science and Technology, Sichuan Agricultural University, Chengdu, Sichuan China; 3 Faculty of Animal Science and Technology, Yunnan Agricultural University, Kunming, Yunnan China; 4 Institute of Animal Nutrition, Sichuan Agricultural University, Chengdu, Sichuan China; 5 Animal Breeding and Genetics Key Laboratory of Sichuan Province, Sichuan Animal Science Academy, Chengdu, Sichuan China; 6 Beijing Institute of Genomics, Chinese Academy of Sciences, and China National Center for Bioinformation, Beijing, China; 7 College of Animal Science and Technology, Northwest A&F University, Yangling, Shaanxi China

**Keywords:** Chromatin structure, Nuclear organization, Transcriptomics

## Abstract

Liver development is a complex process that is regulated by a series of signaling pathways. Three-dimensional (3D) chromatin architecture plays an important role in transcriptional regulation; nonetheless, its dynamics and role in the rapid transition of core liver functions during development and obesity-induced metabolic stress remain largely unexplored. To investigate the dynamic chromatin architecture during liver development and under metabolic stress, we generated high-resolution maps of chromatin architecture for porcine livers across six major developmental stages (from embryonic day 38 to the adult stage) and under a high-fat diet-induced obesity. The characteristically loose chromatin architecture supports a highly plastic genome organization during early liver development, which fundamentally contributes to the rapid functional transitions in the liver after birth. We reveal the multi-scale reorganization of chromatin architecture and its influence on transcriptional regulation of critical signaling processes during liver development, and show its close association with transition in hepatic functions (i.e., from hematopoiesis in the fetus to metabolism and immunity after birth). The limited changes in chromatin structure help explain the observed metabolic adaptation to excessive energy intake in pigs. These results provide a global overview of chromatin architecture dynamics associated with the transition of physiological liver functions between prenatal development and postnatal maturation, and a foundational resource that allows for future in-depth functional characterization.

## Introduction

The liver is an essential, multifunctional, solid organ in mammals that undergoes a rapid transition in its functions during development^1,2^. The fetal liver is committed to hematopoiesis, as hematopoietic stem cells migrate from the yolk sac to the liver^3,4^. Subsequently, its role in hematopoiesis is replaced by the bone marrow. After birth, the liver becomes the metabolic hub for nutrient homeostasis and drug detoxification by coordinating the synthesis, storage, breakdown, and redistribution of nutrients, and by metabolizing xenobiotics^5–7^. The liver plays major immunological and clotting roles during adulthood, and responsible for the production of complement components, cytokines, and chemokines, as well as clotting factors and related inhibitors^8–10^. Liver malfunction due to environmental or/and genetic factors can thus lead to several hepatic disorders, such as non-alcoholic fatty liver disease (NAFLD), which is the most common form of liver disease^11–14^.

Substantial research efforts have been made to characterize changes in the physiological and pharmacological liver functions during development and disease, especially through multidimensional omics approaches, including transcriptomics^15–21^, proteomics^22–24^, and metabolomics profiling^25–28^. A series of critical signaling events—including WNT, FGF, TGF-*β*, and Hippo pathway activation^17,24^—and transcription factors—including HNF4α, ONECUT2, and PROX1^29,30^—underlying liver development and core hepatic functions have been recently identified in humans and rodents.

The mammalian genome is organized in hierarchical layers that enable accessibility to a suite of genes, with rapid rearrangements to accommodate responses to developmental or environmental stimuli^31,32^, including chromosome territories, compartments, and topologically associating domains (TADs), among others^33–35^. In particular, the long-range interaction of promoters with distal regulatory loci is essential for gene regulation by mediating regulatory programs between networks that often span hundreds of kilobases^36,37^. Nevertheless, a panoramic view of the dynamic changes in chromatin architecture underpinning the transitions in liver functions during development and metabolic stress has not yet been thoroughly characterized.

**Fig. 1:**
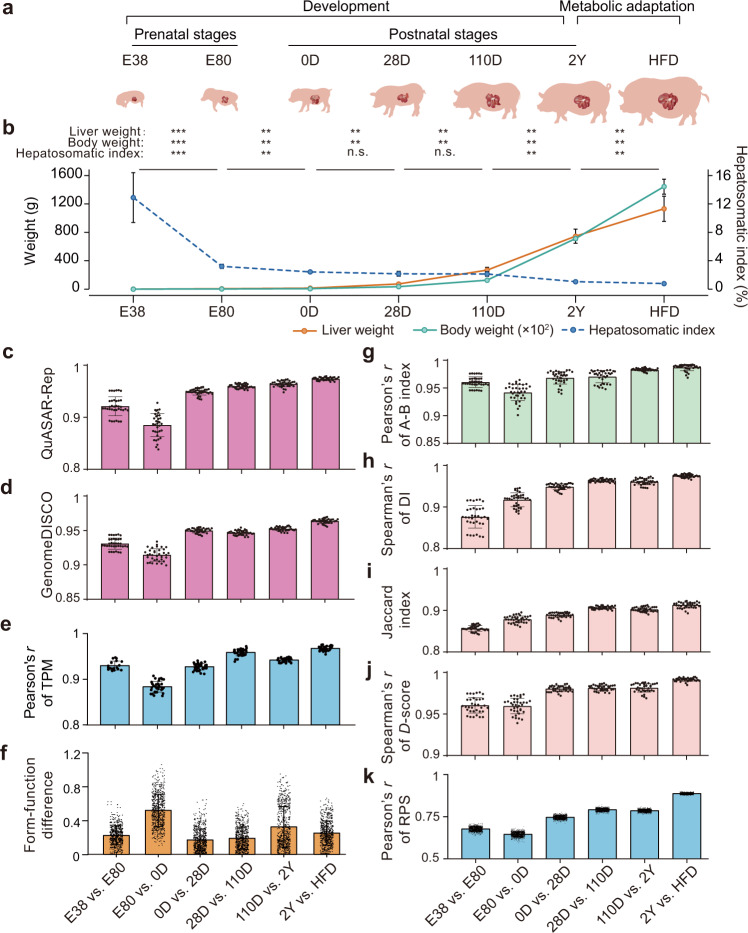
Divergences in chromatin architecture and gene expression of liver during development and metabolic stress. **a** Schematics of liver sampling. **b** Comparison of the hepatosomatic index (ratio of liver to body weight) across six developmental stages and the HFD-fed pigs. Data are presented as means ± SD. *P*-values were calculated using a Wilcoxon rank-sum test. n.s., *P* ≥ 0.05; **P < 0.01; ***P < 0.001. Notably, a rapid liver growth during prenatal development (i.e., greater proportional gain in liver weight compared to body weight) could be observed. **c**–**h** Similarities in chromatin architecture and gene expression for livers between consecutive developmental stages and HFD-fed pigs. The correlations of chromatin architecture were separately determined using **c** QuASAR-Rep and **d** GenomeDISCO for the Hi-C maps; **g** A-B index and **h** Directionality Index (DI) for 20-kb genomic bins; **i** Jaccard index for TADs; **j** *D*-score for consensus TADs (cTADs); and **k** regulatory potential score (RPS) for genes. The dots on the bars represent the replicates. The correlations between gene expression (determined by RNA-seq, **e**) and the combined differences (reflected by the Euclidean distances, **f**) in chromatin architecture (i.e., form) and gene expression (i.e., function) were also measured as described in the Materials and methods.

The domestic pig (*Sus scrofa*) is emerging as a biomedical model that is highly relevant for the study of many complex diseases due to its anatomical, genetic, physiological, and metabolic similarities to humans^38–40^. Here, we employed Bama miniature pigs (an indigenous Chinese breed) as a model to study liver development and metabolic stress. Specifically, we generated high-resolution chromatin contact maps for livers using in situ high-throughput chromatin conformation capture (Hi-C) sequencing across six major developmental stages and under metabolic stress of high-fat diet (HFD)-induced obesity. This experimental setting allowed us to conduct an integrated analysis of chromatin structural and transcriptomic characterization of the porcine liver associated with the transition in hepatic functions from pre- to postnatal development, and metabolic stress during adulthood (Fig. 1a).

## Results

### Chromatin architecture dynamics during liver development and metabolic stress

To elucidate the multi-scale rewiring of chromatin architecture and its influence on gene expression during liver development and metabolic stress, we used in situ Hi-C to map chromatin contacts on the porcine liver throughout six key developmental stages, specifically: embryonic days (1) 38 [E38] and (2) 80 [E80]; (3) birth [0D]; (4) weaning at 28 days [28D]; (5) sexual maturity at 110 days [110D]; (6) and body maturity at 2 years [2Y]). In addition, we conducted in situ Hi-C experiments for liver tissue of adult pigs that were given an HFD diet for 22 weeks (Fig. 1a). We generated a total of ~29.65 billion valid contacts, a large fraction (~65.01%) of which occurred within chromosomes, and constructed contact maps at a maximum resolution of 800 bp by merging the intra-chromosomal contacts of the six replicates at each developmental stage and under the HFD treatment (Supplementary Fig. S1a–c).

We observed a rapid liver growth during prenatal development, inferred by a drop in the hepatosomatic index (i.e., the ratio of liver to body weight) at the E38 (14.7%) and E80 (3.24%) stages compared to the postnatal stages (1.11%–2.45%) (Fig. 1b). Accompanying these changes, correlation analysis for Hi-C maps at 100 kb resolution with QuASAR-Rep^41^ and GenomeDISCO scores^42^ indicated more dramatic shifts in chromatin architecture between the two prenatal stages (QuASAR-Rep score of E38 vs. E80 = 0.92) than observed between neighboring postnatal stages (average QuASAR-Rep score ≥ 0.95) (Fig. 1c, d; Supplementary Fig. S2a, b, i, j). If we focus on consecutive stages, the transition between E80 and 0D exhibited relatively higher differences in chromatin architecture (QuASAR-Rep score = 0.89) (Fig. 1c). These results suggest that the core liver function undergoes more profound changes during prenatal development, whereas following birth these functions stabilize and the liver matures in a more gradual fashion. We also noted that global reprogramming of chromatin architecture in the liver as a response to HFD-induced obesity (QuASAR-Rep score of 2Y vs. HFD = 0.97) was relatively lower than during development (Fig. 1c). The transcriptomic variations (Fig. 1e; Supplementary Fig. S2c, k), the combined differences in chromatin architecture and gene expression (i.e., form-function differences) estimated using a chromosome phase portrait approach^43^ recapitulated these findings (Fig. 1f; Supplementary Fig. S3). This further implies that chromatin architectural changes may facilitate concomitant shifts in transcriptional activity.

### Dispersed chromatin architecture in early liver development

**Fig. 2:**
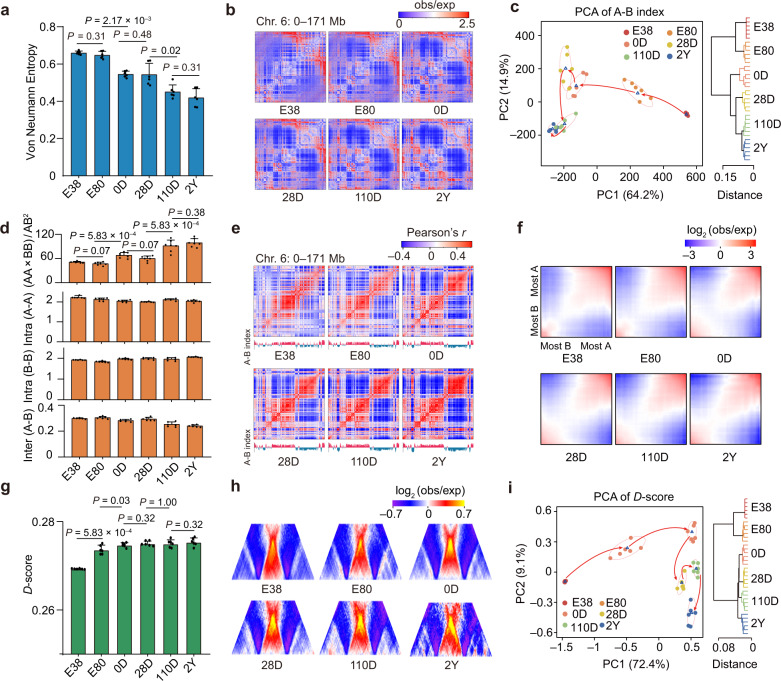
Global reorganization of hierarchical chromatin structures during liver development. **a** Quantification of the disorder in chromatin structure using Von Neumann Entropy (VNE). Higher entropy corresponds to more disordered chromatin structures. Data are shown as means ± SD. The dots on the bars represent the replicates (*n* = 6). **b** Representative observed/expected (O/E) contact maps (100 kb resolution) for chromosome 6. **c** PCA (left) and unsupervised hierarchical clustering (right) of A-B index (*n* = 113,298 bins). Blue triangles indicate the centers of minimum volume ellipses. **d** Changes in compartmentalization strength (calculated as AA × BB/AB^2^) during development. AA, BB, and AB indicate interaction strength within compartments A, B, and between A and B, respectively. Data are shown as means ± SD. The dots on the bars represent the replicates (*n* = 6). **e** Representative correlation matrices (100 kb resolution) and A-B index for chromosome 6. **f** Contact enrichment show the extent of compartmentalization in different stages. **g** *D*-score of cTADs in each stage. Data are shown as means ± SD. The dots on the bars represent the replicates (*n* = 6). **h** Average TAD representation in each stage. An enrichment in the amount contacts was observed during development. **i** PCA (left) and unsupervised hierarchical clustering (right) of *D*-scores for cTADs.

To understand how chromatin architecture shifted during development, we measured changes in 3D structural order across the different stages using the Von Neumann Entropy (VNE) index of multivariate entropies^44^, and observed a gradual decrease in entropy status within chromosomes from E38 to 2Y (mean of 0.66–0.42) (Fig. 2a). This likely reflects a more disordered (the high-entropy status) and relaxed chromatin architecture at early development (E38 and E80) (Fig. 2b). The gradual decrements of inter-chromosomal spatial distances and nuclear radius (reflected by the average distance to the nuclear center of mass) in 3D genome structures during liver development (Supplementary Fig. S4) also support loose (and permissive) chromatin folding during early development. In agreement with the phenomenon that 3D structure in early mammalian embryos is initially obscure but gradually established throughout development^45–47^, the relatively loose chromatin folding highlights a highly plastic state for hepatocyte genomes at the early stages of development and may be essential for the rapid functional transitions in the liver before and after birth.

We subsequently explored the reorganization of the 3D structure at the sub-chromosome level. Based on contact maps at 20-kb resolution, we recognized ~48.68%–57.53% of the genome (~1.10–1.30 Gb in length) as accessible A compartments. These regions exhibited higher levels of GC content, gene density, and transcriptional activity. In contrast, the remaining genome was categorized as less accessible or B compartments, corresponding to 42.47%–51.32% of the genome (~0.96–1.16 Gb) that is characterized by GC-poor, gene-sparse, and transcriptionally inactive (Supplementary Fig. S5a, b). The overall similarity in compartmentalization across the different stages aligned well with the observed similarities in gene expression profiles (Fig. 1e, g; Supplementary Fig. S2c, d, k, l), and revealed a stage-dependent trajectory for compartmentalization during development (Fig. 2c). Notably, we observed that the compartmentalization strength (AA × BB/AB^2^) gradually increased between E38 and 2Y (51.06–99.10) (Fig. 2d). This observation was evident by the reduction in inter-compartment contacts in later stages (median A-B interaction strength of E38 vs 2Y: 0.30 vs 0.24, *P* = 2.17 × 10^–3^, Wilcoxon rank-sum test), and supported by the increasing number of contacts within the B compartment (median B-B interaction strength of E38 vs 2Y: 1.92 vs 2.06, *P* = 2.17 × 10^–3^, Wilcoxon rank-sum test) (Fig. 2d–f).

At a finer scale, we partitioned the genome into 3470–3991 TADs (median sizes of 460–480 kb) (Supplementary Fig. S5c). Although TAD boundaries were largely invariant during development (Spearman’s *r* of DI > 0.88, Jaccard index > 0.86) (Fig. 1h, i; Supplementary Fig. S2e, f, m, n), the ‘connectivity’ (i.e., the tendency for self-interaction) within a given TAD varied and tended to increase over successive stages (median *D*-score of E38 vs 2Y: 0.27 vs 0.28) (Figs. 1j, 2g, h; Supplementary Fig. S2g, o). Principal component analysis (PCA) of *D*-score^48^ kinetics revealed a developing trajectory of intra-TAD connectivity similar to that of compartmentalization (Fig. 2c, i). As expected, *D*-scores positively correlated with gene expression and A compartments (Supplementary Fig. S5d, e). These findings indicate that higher-order chromatin organization gradually solidifies during development.

### Developmental changes in local spatial context affect gene expression

**Fig. 3:**
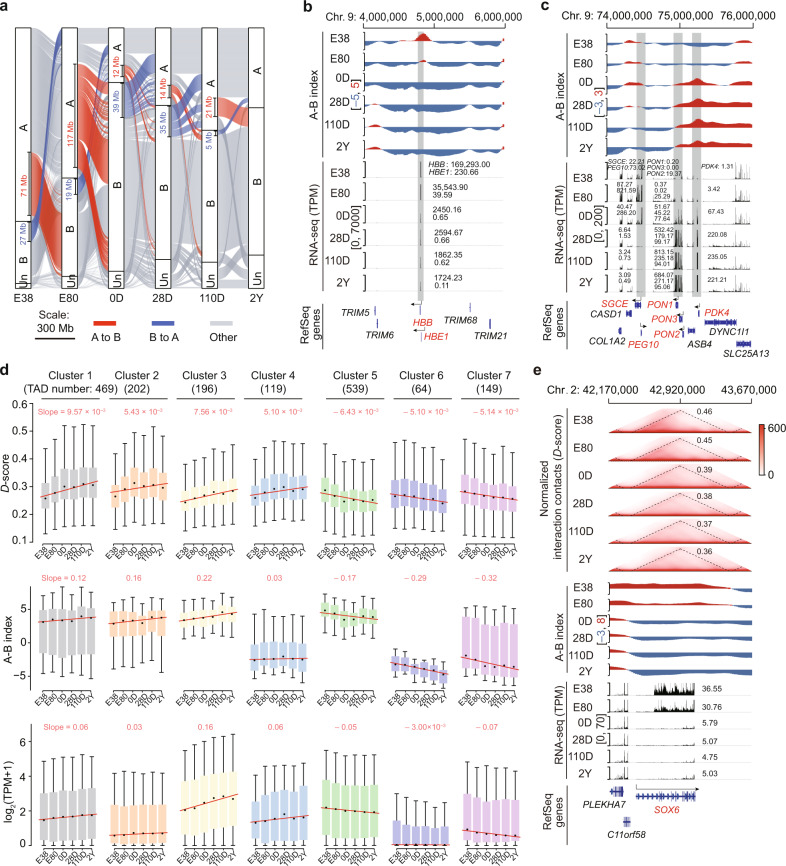
Gene expression affected by shifts in chromatin architecture during liver development. **a** Alluvial representation plot of compartment switching during development. Boxes indicate the length of the genomic regions. ‘Un’ represents genomic regions with uncategorized compartmental status. **b,** **c** Dynamic compartment status and corresponding gene expression for *HBB* and *HBE1* (**b**) and *SGCE*, *PEG10*, *PDK4, PON-1*, *-2*, and *-3* (**c**). **d** Varying TAD connectivity largely coincided with changes in compartmentalization and gene expression during development. Among the seven TAD clusters with developmental stage-dependent *D*-score changes, we found that clusters 1–4 and 5–7 were gradually elevated or reduced, respectively. The slopes of the fitted regression lines are also shown. **e** Representative TADs (containing *SOX6*) with reduced *D*-scores during development.

To explore the functional implications of chromatin architecture shifts during development, we surveyed the genes located in switched compartments and changing TADs between successive developmental stages. We observed substantial levels of compartment switching by genomic regions during development, ranging from ~26.82 Mb (0.19% of the genome) between 110D and 2Y, to ~135.92 Mb (2.05% of the genome) between E80 and 0D (Fig. 3a). These switches in the local spatial context were accompanied by a concomitant increase or decrease in gene expression within regions that respectively switched from B to A or A to B compartments between the successive stages (Supplementary Fig. S6a).

We next investigated possible developmental scenarios for genes undergoing compartmental rearrangement and identified four predominant patterns associated with 2047 genes that are located in compartment-switching regions between two successive stages using the maSigPro-GLM algorithm^49^ (Supplementary Fig. S6b). We found a total of 1535 A compartment genes (659 and 876 genes in clusters 1 and 2, respectively) in prenatal stages that gradually switched to the B compartment in postnatal stages. Functional enrichment analysis using Metascape^50^ suggested that these genes are mainly involved in processes of primary hematopoietic roles in the liver during early development (such as ‘gas transport’, ‘regulation of BMP signaling pathway’, and ‘centrosome cycle’) (Supplementary Fig. S6b). Typical genes essential for hematopoiesis that switched from the A to the B compartment include *HBB*, *HBE1*, *RHAG*, and *SPTA1* (Fig. 3b; Supplementary Fig. S7a, b and Table S1 for functional annotation of the genes), while *CDK1*, *CENPW*, *HMGB2*, *PEG10*, and *SGCE* are critical for liver cell proliferation and embryo development (Fig. 3c; Supplementary Fig. S7c–e and Table S1).

In contrast, a total of 512 genes (170 and 342 genes in clusters 3 and 4, respectively) were identified in B compartments during early stages and gradually switched to A compartments in later stages. These genes are involved in nutrient homeostasis processes (e.g., ‘lipid catabolic process’ and ‘monocarboxylic acid metabolic process’) and xenobiotic metabolism (e.g., ‘response to xenobiotic stimulus’), possibly reflecting the rapid conversion of the liver function in postnatal stages associated with the response to a variety of external stimuli and environment (Supplementary Fig. S6b). Typically, genes participating in the metabolism of glucose, lipids, and amino acids (such as *GYS2*, *PAH*, and *PDK4*), as well as genes associated with drug metabolism (such as *PON-1*, *−2*, and *−3*) gradually shifted to compartment A after birth (Fig. 3c; Supplementary Fig. S7f, g and Table S1). The genes switching to compartment A in the later stages were also enriched for categories associated with growth (e.g., ‘developmental growth’) and immune processes (e.g., ‘positive regulation of natural killer cell chemotaxis’), including two well-characterized growth factor genes (*GHR* and *IGF1*) and several inflammatory indicator genes (*CCL5*, *CCL14*, and *IL1R1*) (Supplementary Fig. S7h–k and Table S1). This is consistent with the rapid growth of the body and enhanced metabolic and immune functions of the liver after birth^1^.

To further determine whether changes in epigenetic chromatin state-mediated compartmentalization occur coincidently with local changes in chromatin accessibility, we performed ATAC-seq assay to measure the differences in local accessibility between the prenatal and adult stages (i.e., E80 vs 2Y) (Supplementary Fig. S8a–e). As expected, we observed that A compartments had higher chromatin accessibility than B compartments, and the stage-specific ATAC peaks mainly occurred in stage-restricted compartment A regions (Supplementary Fig. S8f–h). Supporting the dynamic transition of core liver functions between prenatal development and postnatal maturation^2,18,23^, we found the prenatal E80-specific peaks were enriched in motifs corresponding to the GATA transcription factor family (GATA-1 through -6), of which GATA-1, -2, and -3 are known to be involved in hematopoiesis, while GATA-4, -5, and -6 play roles in endoderm developments^51^ (Supplementary Fig. S8i). In contrast, adult 2Y-specific peaks were enriched in motifs corresponding to seven members of the evolutionarily conserved FOX transcription factor family that regulate diverse biological processes both during development and in the adult, including metabolism (typically, FOXC2, FOXJ3, and FOXL1) and immune (typically, FOXC1, FOXD2, and FOXD3)^52–54^ (Supplementary Fig. S8i).

We next focused on changes in the interaction frequency within TADs during development and identified two representative patterns based on TAD connectivity (determined by *D*-scores, Supplementary Fig. S6c) using MaSigPro^49^. Notably, varying TAD connectivity largely coincided with intra-TAD changes in compartmentalization and transcriptional levels over successive stages (Fig. 3d). More specifically, we found 986 TADs (clusters 1–4) exhibited higher connectivity, which is concordant with the gradually increased compartment scores (i.e., the larger A-B index value) and the upregulation of transcription in these domains throughout development. In contrast, 752 TADs (clusters 5–7) displayed decreasing intra-TAD interactions, compartment scores, and transcription levels (Fig. 3d). The functional categories enriched with genes located in these TADs (Supplementary Fig. S6d) were generally consistent with the transition of liver functions during development and reflected compartmental reorganization, as described above (Supplementary Fig. S6b). For example, *LIN28B* (which is responsible for maintaining embryonic stem cell pluripotency by suppressing the miRNA *let-7*)^55^ and *SOX6* (which enhances erythroid cell development)^56^ were both embedded in low-connectivity TADs and thus decreased in transcription and switched from A to B compartments after birth (Fig. 3e; Supplementary Fig. S7l and Table S1).

Taken together, the reprogramming of hierarchical chromatin architectures, including compartments and TADs, during development likely facilitates the transcription of essential genes required for liver development and function.

### Global rewiring of spatial regulatory circuitry during liver development

Since the 3D physical interactions of promoters and their long-range interacting elements (typically enhancers) dynamically regulate gene expression in a developmental stage-specific manner^36^, we sought to compile an extensive genome-wide catalog of interactions between gene promoters and enhancers (PEIs) throughout six stages of liver development. This analysis revealed 32,557–42,074 PEIs for each stage using the PSYCHIC algorithm^57^ (Supplementary Fig. S9a–e). The analysis was based on ultra-deep contact maps at 5-kb resolution generated by merging the Hi-C contacts of six replicates.

We found that ~80.30% of genes engaged in physical contact with one or more enhancers, and thus tended to be more actively transcribed than those showing no enhancer interactions (Supplementary Fig. S9f). As expected, genes that interacted with more enhancers during development also exhibited higher expression (Supplementary Fig. S9f), which suggested that enhancers provide an additive effect on target gene transcription^58–60^. Accordingly, and in order to accurately elucidate the dynamic rewiring of PEIs during development, we quantitatively explored the regulatory effects of multiple enhancers on individual genes. To this end, we calculated regulatory potential scores (RPSs) for each gene. We found that genes with larger RPS had higher expression (Supplementary Fig. S9g, h), which confirmed the contribution and additive effects of enhancers to increase gene expression^61^. Consistent with findings that showed changes in compartmentalization and TAD connectivity, the overall similarity of PEIs also changed concordantly with that of gene expression over successive stages (Fig. 1k; Supplementary Fig. S2h, p).

Beyond the spatial proximity between enhancers and gene promoters, we analyzed the distribution of H3K27 acetylation (H3K27ac) and H3K4 tri-methylation (H3K4me3) to distinguish the respective effects of enhancers from that of promoters on transcriptional activity^62–64^ using ChIP-seq data (Supplementary Fig. S1e). This enabled a comprehensive dissection of the PEI network rewiring and their regulatory roles during development. Compared to enhancers depleted in H3K27ac peaks, known as poised-enhancers (PEs, 42.21%–60.88% of PEIs), enhancers exhibiting H3K27ac signals were identified as moderately active regular-enhancers (REs, 21.28%–30.72% of PEIs). In addition, enhancers covered by strong H3K27ac signals were highly active super-enhancers (SEs, 11.55%–36.51% of PEIs, having broad acetylation peaks) (Supplementary Fig. S9i, j). As expected, the genes contacting SEs showed higher RPS and had increased expression levels compared with those contacting REs or PEs (Supplementary Fig. S9k, l). An investigation of promoter activity showed that H3K4me3-marked promoters with characteristically elevated transcriptional activity (Supplementary Fig. S9m), preferentially interacted with higher-activity enhancers (i.e., SEs and REs, Supplementary Fig. S9n). In contrast, inactive promoters that were absent from H3K4me3 peaks were generally accompanied by less active enhancers (i.e., PEs) (Supplementary Fig. S9n). To confirm the reliability of enhancers identified here, we randomly selected 2–10 enhancers of three genes (identified in 2Y) for validation in HEK-293T cells using the Dual-Luciferase reporter assay. The results showed significantly increased transcriptional activities for most of the tested enhancers compared with the controls (*P* < 0.05, two-sided Student’s *t*-test, Supplementary Fig. 9o and Table S2). These findings support the remarkable role of enhancer activity in transcription control^62^.

**Fig. 4:**
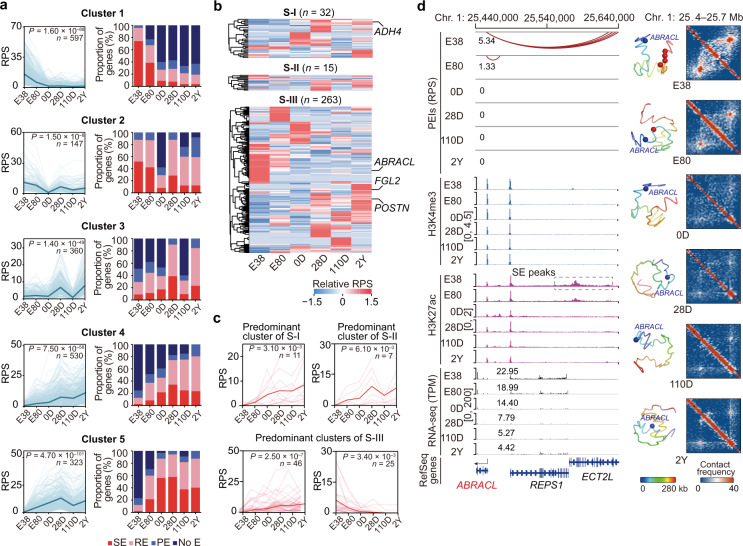
Developmental-dependent changes of PEI-associated transcriptional regulation. **a** Developmental-dependent changes in gene RPS (|log_2_FC | > 2 and |ΔRPS | > 3) between consecutive stages were classified into five clusters using the STEM algorithm (left). The bold lines indicate the mean RPS, and the thin lines represent the RPS of each gene in the relevant cluster during development. FDR-corrected *P*-values were obtained from multiple hypothesis testing. The proportion of genes that interacted with differently active enhancers (activity ranking: super-enhancer (SE) > regular-enhancer (RE) > poised-enhancer (PE)) at each stage were consistent with the dynamic RPS (right). **b** Heatmaps showing temporal RPS patterns of the signature genes in three HCC subtypes (S-I, S-II, or S-III) across stages. Genes were grouped using hierarchical clustering. Four signature genes are labelled on the plot. **c** Predominant RPS patterns of signature genes for three HCC subtypes identified using the STEM algorithm. **d** PEI rewiring of *ABRACL*, a signature gene in S-III HCC. Left: Schematics of PEIs, H3K4me3, and H3K27ac signals, and transcription. Dashed boxes highlight super-enhancer (SE) peaks. Right: 3D structural models and Hi-C contact maps of the corresponding genomic region (seven 5 kb bins up- and downstream from the bin containing the transcription start site (TSS)). Gene promoters and enhancers are shown as blue and red spheres in the 3D models, respectively.

Next, we identified five representative patterns of genes exhibiting developmental-dependent changes in RPS (Fig. 4a) using the STEM algorithm^65^. These five patterns were then classified according to the presence of higher RPS before birth compared to after birth (597 and 147 genes in clusters 1 and 2, respectively) or vice versa (360, 530, and 323 genes in clusters 3, 4, and 5, respectively). Functional enrichment analysis showed that genes in the former group are enriched for processes including ‘myeloid cell differentiation’, ‘gas transport’, and ‘erythrocyte homeostasis’, reflecting the hematopoietic function of the fetal liver (Supplementary Fig. S10a). In contrast, genes in the latter group are mainly involved in metabolic processes such as ‘lipid biosynthetic process’ and ‘carboxylic acid biosynthetic process’, coinciding with the known metabolic functions of the liver after birth (Supplementary Fig. S10a). Furthermore, we found that genes with high, stage-specific RPS were more likely to interact with active enhancers (SEs or REs) (Fig. 4a). For example, the 26 genes present in cluster 3 that are enriched for ‘monocarboxylic acid metabolic process’, are mostly regulated by REs or SEs after birth (Supplementary Fig. S10b).

Supporting the transcriptional similarity between liver development and the human hepatocellular carcinoma (HCC)^66^, more aggressive tumors have an aberrant reactivation of some developmental processes (typically, cell proliferation) that need to be silenced in the adult liver. In turn, less aggressive tumors typically maintain a series of well-orchestrated metabolic events that are elevated in the mature liver^23^. We observed that the predominant developmental patterns in RPS of 32 signature genes for the less aggressive HCC subtype (S-I, good prognosis) gradually increased after birth (11 genes; typically, *ADH4*) (Fig. 4b, c; Supplementary Fig. S10c, Table S1 and Data S1). In contrast, a substantial proportion of the 263 signature genes linked to the more aggressive HCC subtype (S-III, poor prognosis) exhibited increased RPS either after birth (46 genes; typically, *FGL2* and *POSTN*, both involved in leukocyte activation) or during early development (25 genes; remarkably, the cell proliferation marker, *ABRACL*) (Fig. 4b–d; Supplementary Fig. S10d, e, Table S1 and Data S1).

### Rewiring of PEIs underpinning functional transition in the liver during development

To further explore PEI reorganization associated with shifts in core liver functions throughout development (hematopoiesis in the fetus, metabolism, and immunity after birth), we examined changes in RPS profiles for eight a priori representative candidate gene sets (Supplementary Data S2).

**Fig. 5:**
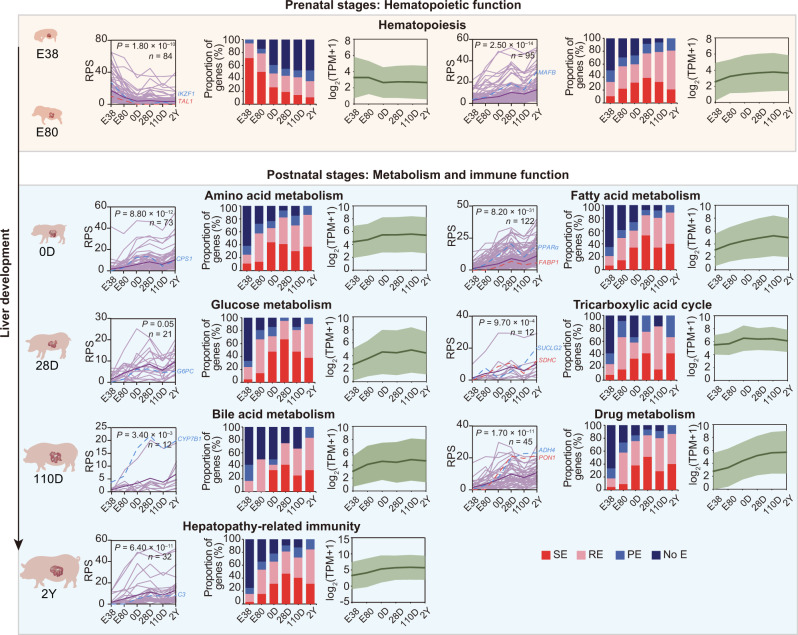
Rewiring of PEIs associated with shifts in core liver functions during development. The predominant RPS patterns across developmental stages were separately identified for eight a priori gene sets associated with core liver functions at prenatal (hematopoiesis) and postnatal stages (metabolism of amino acid, fatty acid, glucose, bile acid, and drug; tricarboxylic acid cycle; and immunity) (Supplementary Data S2) using STEM. The bold lines in the left panels represent the mean RPS, and the thin lines represent the RPS of each gene in the relevant cluster during development. FDR-corrected *P*-values were obtained from multiple hypothesis testing. For each RPS cluster, the proportions of genes that interacted with differently active enhancers (activity ranking: SE > RE > PE) at each stage (middle) and the gene expression changes (right) were consistent with the dynamic RPS (left). Representative functional genes are labelled on the plot.

**Fig. 6:**
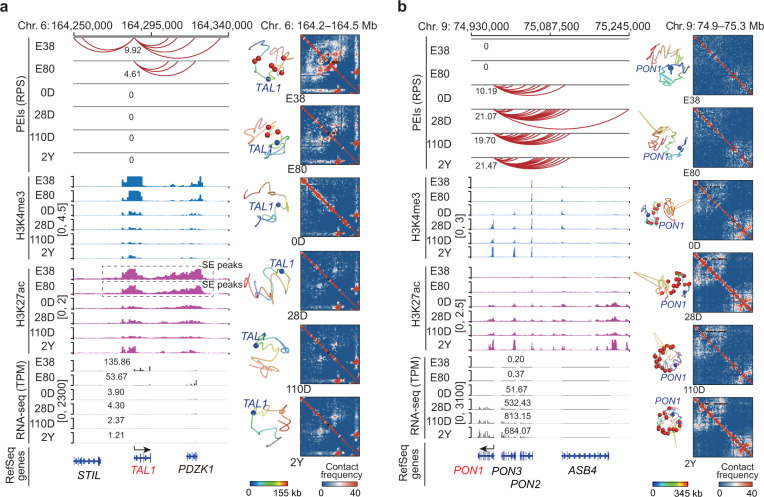
Examples of PEI rewiring related to transition of liver functions during development. **a** *TAL1* and **b** *PON1* respectively participate in hematopoiesis and drug metabolism. Left: schematics of PEIs, H3K4me3, and H3K27ac signals, and transcription. Right: 3D structural models and Hi-C contact maps of the corresponding genomic regions. Gene promoters (blue spheres) and enhancers (red spheres) are shown.

Strikingly, we identified two significant RPS profiles during development among the 719 hematopoietic genes. One predominant pattern involved 84 genes that showed a gradual decrease in RPS over successive stages (Fig. 5; Supplementary Fig. S11). Fifty-four (or 64.29%) of these genes were associated with the ‘myeloid cell differentiation’ process (Benjamini-Hochberg *P* < 10^–16^, hypergeometric test). This result supports the critical role of hematopoiesis in fetal liver^3^. As with most of these genes, *TAL1* and *IKZF1* interacted with SEs at E38 and E80 but lost all enhancer contacts after birth (Fig. 6a; Supplementary Fig. S12a, Table S1 and Data S2). In contrast, the other predominant pattern involved 95 genes that showed a gradual increase in RPS over successive stages. Among these, 55 (or 57.89%) were involved in ‘leukocyte differentiation’ (Benjamini-Hochberg *P* < 10^–16^, hypergeometric test), which supports the well-established substantial increase in immunologically active cells in the postnatal liver. For example, *MAFB* only interacted with PEs at E38, but interacted with REs at E80 and SEs after birth (Supplementary Fig. S12b and Data S2). These two distinct patterns in RPS profiles are consistent with a diminished hematopoietic capacity and improved immune function in the postnatal liver^23^.

Notably, we found that six metabolic gene sets (metabolism of amino acid, fatty acid, glucose, bile acid, and drug, as well as tricarboxylic acid cycle) and hepatopathy-related immune genes predominantly exhibited a gradual increase in RPS during development (*P* ≤ 0.05, multi-hypothesis test) (Fig. 5; Supplementary Fig. S11). This strongly supports the increasing importance of metabolism and immunity for the liver after birth^23^. Genes with increased RPS profiles included *CPS1* (amino acid metabolism), *FABP1* and *PPARα* (fatty acid metabolism), *G6PC* (glucose metabolism), *SDHC* and *SUCLG2* (tricarboxylic acid cycle), *CYP7B1* (bile acid metabolism), *ADH4* and *PON1* (drug metabolism), and *C3* (innate immune). All of these genes showed little to no prenatal enhancer contacts, but were upregulated and had increased enhancer interactions (e.g., with SEs) following birth (Fig. 6b; Supplementary Figs. S10c, S12c–j, Table S1 and Data S2).

### Chromatin architecture shifts in the liver responding to HFD-induced obesity

The liver plays a central role in metabolic homeostasis after birth^2^ but may suffer from various disorders due to metabolic stress, of which the most prevalent is NAFLD caused by high caloric input-induced obesity^67^. To investigate 3D genome responses to metabolic stress in the liver, we performed a multi-scale comparison of chromatin architectures with related phenotypes between the livers of pigs fed with HFD and pigs fed normal adult diets.

**Fig. 7:**
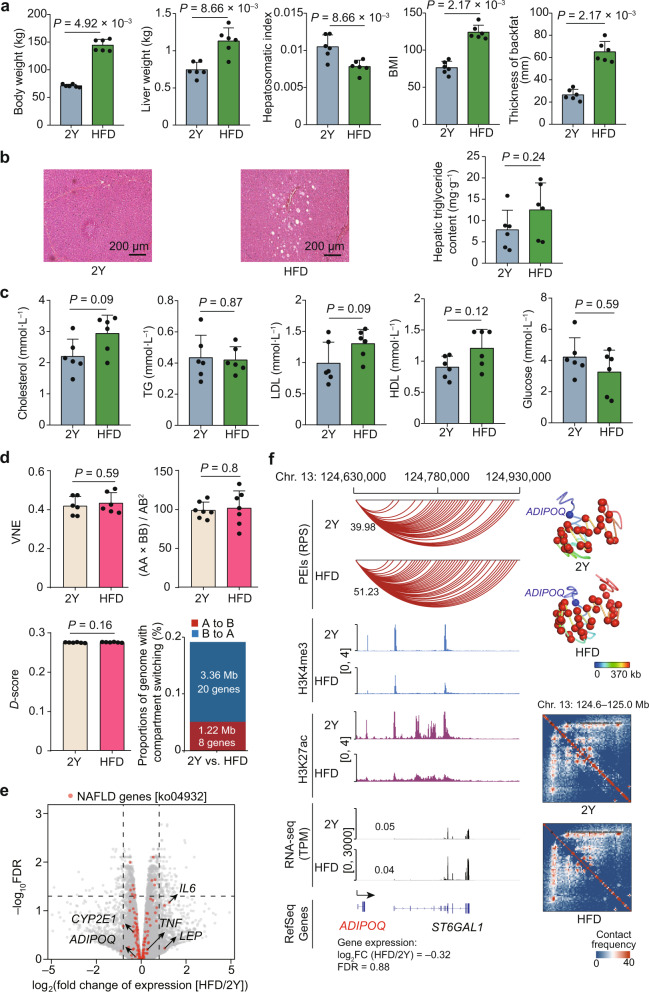
Changes in phenotypes related to hepatic function, chromatin architecture, and gene expression as a response to liver metabolic stress in pigs. **a** Comparison of obesity-related phenotypes. **b** Representative H&E staining of paraffin sections and measurement of triglyceride contents in the liver. Data represent means ± SD. **c** Serum concentrations of five metabolic indicators. TG triglyceride, LDL low-density lipoprotein, HDL high-density lipoprotein. **d** Subtle alterations in chromatin architecture, reflected by VNE, compartmentalization strength, intra-TAD connectivity, and compartment switching. **e** Transcriptomic comparison between HFD- and normal diet-fed pig livers. Red dots represent the 126 NAFLD-related genes obtained from the KEGG pathway (ko04932). Five typical NAFLD markers are indicated. **f** Schematic representation of PEIs, 3D structural models, and Hi-C contact maps for *ADIPOQ*. Left: a schematic representation of PEIs, H3K4me3, and H3K27ac signals, and transcription. Right: 3D structural models and Hi-C contact maps of the corresponding genomic regions. Gene promoters and enhancers are shown as blue and red spheres, respectively.

As expected, HFD feeding for 22 weeks resulted in a dramatic increase in liver weight (fold change = 1.51, *P* = 8.66 × 10^–3^, Wilcoxon rank-sum test), body weight (fold change = 2.03, *P* = 4.92 × 10^–3^), backfat thickness (fold change = 2.46, *P* = 2.17 × 10^–3^), and body mass index (BMI) (fold change = 1.62, *P* = 2.17 × 10^–3^) (Fig. 7a). All of the above represent strong indicators of obesity. Nonetheless, the hepatosomatic index (fold change = 0.75, *P* = 8.66 × 10^–3^) decreased in the HFD-fed pigs. At the same time, histological evaluation of liver sections showed almost normal hepatic morphology, minimal inflammation, and a non-significant increase in lipid accumulation (hepatic triglyceride content for normal diet vs HFD, 7.82 vs 12.51, *P* = 0.24, Wilcoxon rank-sum test) in the enlarged livers of HFD-fed pigs (Fig. 7b). Moreover, no statistically significant differences were observed in the serum concentrations of five metabolic indicators between pigs fed with HFD and normal diets (Fig. 7c).

In agreement with the limited changes in metabolism-related phenotypes, we found subtle but widespread changes in chromatin architecture in the liver of HFD-fed pigs. These changes were much weaker than those observed during development, including high similarity in Hi-C contact maps (Fig. 1c, d; Supplementary Fig. S2a, b, i, j), comparable VNE (Fig. 7d), similar compartmentalization strength (Fig. 7d; Supplementary Fig. S2d, l), largely conserved TAD boundaries (Fig. 1h, i; Supplementary Fig. S2e, f, m, n), and comparable intra-TAD contact intensities (Fig. 7d; Supplementary Fig. S2g, o). In addition, only 0.2% of genome (4.58 Mb) exhibited compartment switching between HFD and normal diet pigs (Fig. 7d).

We have not observed obviously aberrant transcription of genes associated with liver metabolic stress after HFD-induced obesity. Of the 160 genes exhibiting significant expression changes (|log_2_FC| > 1 and FDR < 0.05) between HFD and normal diet-fed pigs (Supplementary Fig. S13a, b), only nine showed concomitant RPS changes (|log_2_FC| > 2 and |ΔRPS|> 3) (Supplementary Fig. S13c). Notably, none of the 126 NAFLD-related genes in the autosomes (retrieved from the KEGG pathway: non-alcoholic fatty liver disease [ko04932]) exhibited significant expression changes between the two groups (Fig. 7e, f; Supplementary Data S3). Five typical NAFLD markers, including *ADIPOQ, CYP2E1*, *IL6*, *LEP,* and *TNF*, also displayed comparable RPS and expression levels between the groups (Fig. 7e, f; Supplementary Fig. S13d–g, Table S1 and Data S3).

We next measured the spatial proximity between promoters, which represents an additional active transcriptional program responding to signaling and environmental cues^36^. Consistent with the previous observations in mouse^68^, human^69^, and pig^70^, genes with relatively high expression exhibited an elevated extent of spatial associations, which were most likely to be occupied by common transcription factors (Supplementary Fig. S14a). Nonetheless, no canonical transcription factors involved in metabolic stress were predicted to preferentially bind at genomic sites of 245 highly expressed genes in the liver of HFD over normally fed pigs (Supplementary Fig. S14a). Consistently, the expression of target genes of HNF4α and C/EBPα (two essential transcription factors participating in the regulation of NAFLD-related metabolism genes)^71^ in the liver of HFD- and normally fed pigs was also comparable (*P* > 0.05, Wilcoxon rank-sum test) (Supplementary Fig. S14b).

No obvious metabolic dysfunction could be found in the liver of HFD-fed pigs (Fig. 7), suggesting domestic pigs may potentially resist NAFLD in spite of obesity. If that is the case, this constitutes a distinctive pattern from humans, who present NAFLD in up to ~70% of overweight individuals^17,72,73^, and rodents, who normally manifest NAFLD when undergoing high-calorie diet-induced obesity (e.g., ~1.2–1.4-fold gain in body weight)^74^. This inter-specific discrepancy indicates that domestic pigs probably developed metabolic adaptions to ‘diabetogenic’ environments (energy abundance and little physical exercise)^75–77^ and are potentially resistant to the chronically deleterious effects of obesity in the liver^78–80^. A similar selection scenario of protective mechanism has also been observed for domestic cats and dogs (as well as human populations in developed countries), which exhibit more superior performance in resistance to metabolic risk factors than rodent-catching cats and hunting dogs (and, human populations in developing countries)^75^.

## Discussion

This study reports the structural dynamics of the 3D genome in a domestic pig model to illustrate how shifts in higher-order chromatin architectures and transcriptomic regulation are closely associated with rapid transitions in liver functions during prenatal development and maturation after birth, or under metabolic stress in adulthood. Multi-scale 3D genome reorganization together with enhancer and promoter activity dynamically regulates gene expression in a well-orchestrated temporal manner.

We found that the earlier stages of development (E38 and E80) showed more relaxed chromatin architecture compared to postnatal stages, which coincide with an expansion of accessible compartment A regions (Supplementary Fig. S15a, b). Compared with compartment B regions, compartment A regions are more gene dense, harbor activating chromatin marks, and have more highly active enhancers and promoters that are necessary for widespread active transcription (Supplementary Fig. S15b, c). The characteristically highly plastic genome organization of earlier developmental stages includes permissive chromatin that allows for the transcription of extensive genomic regions and facilitates rapid functional transition in the liver during development. These observations expand current knowledge on the genetics of liver development and maturation^1^.

It is worth noting that the liver undergoes a substantial change in its cellular composition during embryonic development and growth^18,81,82^, nonetheless, our bulk Hi-C data only provide the average features of chromatin architecture on a cell population scale. To what extent cellular heterogeneity of the liver contributes to the observed differential signals of chromatin features is still required to investigate at a single-cell resolution^83–85^.

Pigs, in particular miniature pig breeds, have recently emerged as an attractive biomedical model for the study of metabolic diseases^39^. Our work greatly expands the annotation of regulatory DNA elements (enhancers) in the reference pig genome. As expected, using the NHGRI-EBI GWAS catalog (https://www.ebi.ac.uk/gwas/) and the LiftOver tool (https://genome.ucsc.edu/cgi-bin/hgLiftOver), we found DNA sequence variation (human noncoding SNPs) associated with specific traits or diseases were enriched in enhancers identified in the porcine liver (mean of SNP enrichment scores in enhancers vs. non-enhancer regions: 1.35 vs 0.88, *P* = 0.014, Wilcoxon rank-sum test). This is more pronounced in the case of SEs (mean SNP enrichment score in SE regions: 2.08) (Supplementary Fig. S16a), confirming the robustness and functional roles of these regulatory elements^86,87^. In support of the findings describing the PEI rewiring associated with shifts in liver functions during development, we observed that hematopoiesis- and metabolism-associated SNPs are separately enriched during pre- and postnatal stages (Supplementary Fig. S16b, c). These genetic results, combined with the great physiological similarities between pigs and humans, provide further support for the use of pigs as an attractive model for studying hepatology in humans.

Importantly, both the 3D genomic and phenotypic data presented in this study showed pigs are to some extent resistant to HFD-induced NAFLD, which provides essential information regarding the application of domestic pigs as a liver disease model. Hence, we corroborate our previous arguments that the evolutionary divergence (particularly, transcriptional shifts of homologous tissues) between pigs and other mammalian models needs to be considered when selecting targets in animal models to extrapolate diseases or traits^39^.

## Materials and methods

### Ethics statement

Animal maintenance and experimental procedures were approved by the Institutional Animal Care and Use Committee in the College of Animal Science and Technology, Sichuan Agricultural University, Sichuan, China under permit No. DKY-2019102015. Throughout the procedure, particular care was taken to avoid animal suffering and to ensure ethical treatment.

### Experimental animals

We used Bama pigs of the similar age and physical condition across six developmental stages, including two prenatal stages—embryonic day 38 (E38; *n* = 14 individuals) and embryonic day 80 (E80; *n* = 6), and four postnatal stages—birth day (0D; *n* = 6), 28 days after birth (28D; *n* = 6), sexual maturity at 110 days (110D; *n* = 6), and body maturity at 2 years (2Y; *n* = 6). We also used pigs from an HFD treatment group (*n* = 6). For the HFD treatment, the pigs were fed a high-fat diet (15.12 MJ/kg metabolizable energy, 11.26% crude protein, 6.8% fat, and 5% lysine) for 22 weeks in order to induce obesity. A total of six pigs at the developmental stage of 2Y were used as normal diet controls (12.9 MJ/kg metabolizable energy, 15.37% crude protein, 2% fat, and 6.7% lysine). All pigs fasted for twelve hours before being sacrificed. Since the sexual characteristics of pig fetuses are not visible before day 49 post-conception, the gender was determined at E38 using a PCR-based method, similar to previously described^88^. Livers were collected immediately after sacrificing the pigs and snap-frozen in liquid nitrogen for subsequent assays. All liver samples at E38 (*n* = 14) were pooled for high-throughput sequencing analysis.

### Phenotypic measurements

We measured liver weight, body weight, and body length of all pigs using conventional methods, and calculated their hepatosomatic index (the ratio of liver weight to body weight) and body mass index (BMI, body weight/body length^2^).

For the 2Y control group and the HFD treatment group, fresh liver tissue was fixed in 10% formalin at room temperature for 12 h, and then dehydrated and embedded with paraffin. Paraffin sections were prepared and stained with an Hematoxylin and Eosin (HE) Staining Kit (C0109 and C0107, Beyotime) according to the manufacturer’s instructions. To measure lipid levels in HFD-fed animals, frozen liver samples from both the HFD treatment and 2Y control groups were ground by mortar and pestle under liquid nitrogen and examined for triglyceride (TG) content with a Triglyceride Content Assay kit (AKFA003C, Boxbio) according to the manufacturer’s instructions. Venous blood (50 mL) was also collected from fasting pigs of both experimental groups. After this, circulating indicators in the serum were measured in each pig using a CL-8000 clinical chemical analyser (Shimadzu) via standard enzymatic procedures.

### In situ Hi-C library preparation and sequencing

We constructed six Hi-C libraries (including six technical replicates for E38 and six biological replicates for each of the other stages and HFD treatment) for livers at each developmental stage and HFD treatment group, as previously described^89^ with minor modifications. Briefly, liver tissue was homogenized with liquid nitrogen and then fixed with a 4% formaldehyde solution at room temperature for 30 min. After fixing, 0.2 mol/L glycine was used to quench the reaction and the samples were suspended in a lysis buffer (1 mol/L Tris-HCl, 1 mol/L NaCl, 10% CA-630, and protease inhibitors) on ice for 15 min. The cell nuclei were lysed with 0.1% SDS and quenched with 0.1% TritonX-100. The chromatin was digested with 200 U DpnII (R0543S, NEB) at 37 °C for 90 min. Next, a total of 0.4 mM Biotin-14-dATP (19524–016, Invitrogen), 10 mM dCTP, 10 mM dGTP, 10 mM dTTP, and 5 U/μL Klenow Fragment were used to fill-in and mark the DNA fragment. For ligation, T4 DNA ligase was added and the samples incubated at room temperature for 4 hours with slow rotation. DNA was purified by ethanol precipitation and then sheared using a probe sonicator to fragment size of ~400 bp. Biotin-tagged DNA fragments were pulled down with M280 beads. The Hi-C libraries were amplified for 10 PCR cycles and sequenced as 100 bp paired-end reads on a BGISEQ-500 platform.

### Hi-C data processing

Hi-C reads were processed using a custom pipeline in Juicer^90^ software (v 1.8.9, https://github.com/aidenlab/juicer/wiki). Briefly, high-quality Hi-C reads (1255.27 million per sample) were aligned to the pig reference genome (Sscrofa 11.1) using BWA^91^ (v 0.7.15, http://bio-bwa.sourceforge.net/) with default parameters. Then, the read pairs that could not be successfully aligned or PCR duplicates were filtered out with Juicer. Low-quality alignments with MAPQ < 30 were removed, too. Finally, intra-chromosomal contract matrices were separately generated at 5, 20, and 100 kb resolutions using valid read pairs, then were normalized using the Knight-Ruiz (KR)^92^ matrix balancing algorithm and quantile algorithms, while 1 Mb inter-chromosomal metrices were generated using the KR^92^ algorithm and log-counts per million (CPM; i.e., the average abundance across all libraries) normalization method.

The correlations between normalized intra-chromosomal matrices were calculated using QuASAR-Rep^41^ and GenomeDISCO^42^ with default parameters. We applied the Von Neumann Entropy (VNE) approach to quantify the order of chromatin organization for 100-kb resolution intra-chromosomal matrices as previously described^44^. Higher entropy corresponds to more disorder. First, correlation matrix C was calculated as C = corr (log_2_[A]), where A represents the Hi-C matrix of each autosome. Second, we conducted eigen-decomposition of matrix C, where λ1 ≤ λi ≤ λn were the eigenvalues of matrix C. The eigenvalues were then normalized: $\lambda i = \frac{{\lambda i}}{{\mathop {\sum}\nolimits_{j = 1}^n {\lambda j} }}$. Finally, VNE was computed as

$${{{\mathrm{VNE}}}} = - \mathop {\sum}\limits_{i = 1}^n {\bar \lambda i{{{\mathrm{ln}}}}(\bar \lambda i)} .$$

Compartments A/B at 20 kb resolution were identified using both principal component analysis (PCA) and A-B index, as previously described^93^. First, PCA was performed to generate PC1 vectors for each autosome per sample at 100 kb resolution. Spearman’s correlations between PC1 and genomic characteristics including gene density and GC content were calculated. Bins with positive Spearman’s correlation were defined as compartments A, whereas the remainder were defined as compartments B. The A-B index was then calculated as previously described^93^ at 20 kb resolution, which represents the likelihood of a genomic segment interacting with the A or B compartments defined at 100 kb resolution as above described. Bins of 20 kb length with positive or negative A-B index were considered as A or B compartments, respectively. The final compartment status of each bin for every developmental stage or the HFD treatment was determined as the consistent status of more than three biological replicates.

For the compartmentalization plot (‘saddle plot’), we rearranged the A-B index of 20 kb bins from the lowest to the highest in each autosome, then reshuffled the observed/expected (O/E) map of this chromosome and divided the resulting map into 50 × 50 sub-matrices. Compartmentalization strength^47,93^ was defined as (AA × BB)/AB^2^, where AA and BB represent the mean levels of interactions between regions of the same compartment status, while AB corresponds to the mean interaction frequency between regions belonging to different compartments.

TADs were identified at 20 kb resolution using Directionality Index (DI)^93,94^ and Insulation Score (IS)^95^. DI was calculated for each 20 kb bin based on the interactions of the ten bins immediately upstream and downstream from the center of each bin using a previously described method^94^. We used a hidden Markov model (HMM) to predict TAD boundaries based on the state of DI. In addition, IS was calculated and normalized for each 20 kb bin following^95^. Minimal IS along the normalized IS vector was interpreted as the TAD boundary. Finally, large TADs identified by DI were further split into small TADs based on IS, and then two sets of TADs were merged for further analyses. Jaccard Index and Pearson’s correlation of DI were calculated to assess the variation of TAD boundaries across developmental stages as well as between the HFD treatment and normal diet controls.

To quantify intra-TAD interaction strength with Domain score (*D*-score)^48^, we determined consensus TADs (cTADs), which were defined as TADs conserved in at least 50% of the developmental stages and replicates, or those emerging in both HFD and controls. The *D*-score of a cTAD indicated the ratio of intra-TAD interactions across all intra-chromosomal (intra-TAD and inter-TAD) interactions. The A-B index of a cTAD was calculated as the average A-B index of all bins within that cTAD. The compartment status (A, B, or mixed) of a cTAD was determined by the frequency of the A/B status within the cTAD, using 80% as the threshold.

To identify chromatin interactions at the gene level, Hi-C read pairs derived from six technical (for E38 only) or biological replicates were merged to generate 5 kb resolution contact matrices, and over-represented chromatin interactions with gene promoters, known as PEIs, were identified based on the 5 kb matrices using the PSYCHIC^57^ algorithm (https://github.com/dhkron/PSYCHIC) with default parameters. PEIs with high confidence (FDR ≤ 0.01 and interaction distance ≥ 20 kb) were retained. The normalized Hi-C contact matrix was split into smaller matrices (20 × 20 Mb) with a step size of 10 Mb to accelerate computing.

To explore the regulatory effects of multiple enhancers on a gene, and accurately depict the dynamic rewiring of PEIs over the course of development, we measured the regulatory potential score (RPS) for each gene based on the biochemical principle that an enhancer’s regulatory effect on a gene is dependent on its spatial proximity, with multiple enhancers producing an additive effect on the upregulation of target gene transcription. The RPS was calculated as ∑n (log_10_In), where In indicates the normalized interaction intensity (i.e., the observed value minus the expected value). If a promoter interacts with no enhancer, then the RPS equals zero. Since genes with low RPS might have high RPS fold changes (FC) but small RPS fluctuations across developmental stages, we defined differential RPS for genes using the formula |log_2_FC| > 2 and |ΔRPS| > 3. We set the RPS of genes with no enhancers as 0.1 in order to facilitate the calculation of log_2_FC of RPS.

We reconstructed the 3D genome structures with both intra- (20 kb resolution) and inter-chromosomal (1 Mb resolution) contacts using Python package miniMDS^96^ with default parameters. We also visualized the 3D genome using PyMOL (v 2.5.2, https://pymol.org/2/).

### Dual-luciferase reporter assay

The reliability of identified enhancers was verified by a Dual-Luciferase Reporter Assay System^97^. The chromosome coordinates and primer sequences of tested enhancers and promoters are shown in Supplementary Table S2. The genomic DNA of adult pig liver was used as a template to amplify the enhancer and promoter regions with a length of 1–2 kb. The amplified products were separated and purified on an agarose gel. The amplified promoters were cloned into the pGL3-Basic plasmid (Promega, E1751) which was digested with *Kpn*I and *Hind*III and inserted into a linker sequence, termed as pGL3-Promoter plasmid. Then the amplified enhancers were cloned into the pGL3-Promoter plasmid digested with *Sal*i, that is, pGL3-Promoter-Enhancer plasmid. All constructed vectors were verified by sequencing. pGL3-Basic or constructed plasmids were transfected into HEK-293T cells and incubated for 36 h using Lipofectamine 3000 (Invitrogen, L3000015) and Opti-MEM (Gibco, 11058021), with three technical replicates for each construct. And TK-15 (Promega, E2241) was cotransfected for each as a normalization control. Fluorescence values were determined using a Dual-Luciferase Reporter Assay System (Promega, E1960), according to the manual. Relative fluorescence values of each construct were calculated based on the signal of pGL3-Basic plasmid.

### Chromosome phase portrait

To explore the combined differences in chromatin architecture and gene expression profiling (i.e., form and function, respectively) between developmental stages, we implemented a quantitative assessment of form-function dynamics at the chromosome level across all samples at the six stages and under HFD treatment, which resulted in a so-called chromosome phase portrait^43^. The chromosomal architecture was characterized by the network connectivity (Fiedler number, FN) of chromatin contacts^43^. The genomic function was inferred by measuring the expression level (mean of TPM) based on RNA-seq data. The difference in form-function is indicated by the two-dimensional (2D) distance (Euclidean distance) calculated using the two characteristic values.

### RNA-seq library preparation and sequencing

Total RNA was extracted from snap-frozen liver tissue using the RNeasy Mini Kit (Qiagen) according to the manufacturer’s instructions. RNA samples were quantified by Qubit RNA Assay Kit (Q10211, LT) according to the manufacturer’s instructions. The integrity of the RNA was detected using the Agilent RNA 2000 Nano kit.

We constructed six RNA-seq libraries (biological replicates) for each developmental stage (E80, 0D, 28D, 110D, and 2Y) and the HFD-fed group. Three RNA-seq libraries (technical replicates) were constructed for the pooled liver tissues of 14 female fetuses at E38. We used an rRNA depletion protocol (Ribo-Zero kit, Epicentre) coupled with the Illumina TruSeq stranded RNA-seq library protocol to construct the RNA-seq libraries. Briefly, ribosomal RNA (rRNA) was removed from total RNA using the Ribo-zeroTM rRNA Removal Kit (RZH1046, Epicentre). The ribosome-free RNA samples were then purified by ethanol precipitation. Next, NEBNext^®^ UltraTM Directional RNA Library Prep Kit (E7420S, NEB) was used to construct the sequencing library. In short, second-order cations were used to fragment RNA using the NEBNext First Strand Synthesis Reaction Buffer (5×). Single strand cDNA was synthesized using a random six-base primer and M-Mulv Reverse transcriptase (RNase H free). The second strand of the cDNA was synthesized with buffer, dNTPs (i.e., dUTP, dATP, dGTP, and dCTP), DNA polymerase I, and RNase H. After purification, terminal repair, the addition of poly A, and ligation of sequencing joints, the cDNA was purified with the USER enzyme to degrade the cDNA containing uracil (U), and a PCR enrichment was performed. Finally, AMPure XP Beads were used to purify the PCR products and obtain the final library. After this, q-PCR was used to accurately quantify the library concentration. Each RNA-seq library was sequenced as 150 bp paired-end reads using the Illumina HiSeq X Ten platform.

### RNA-seq data processing

After filtering reads containing over 10% of non-determined nucleotides (‘N’) or over 50% of the low-quality score (Q value < 5) bases, a total of 39 RNA-seq libraries were generated at an average of 43.46 million 150 bp paired-end high-quality reads for each library (Supplementary Fig. S1d). High-quality reads were aligned to the pig genome (Sscrofa 11.1) using STAR (v 2.5.0a)^98^ in a basic two pass mode using the “Encode” option as specified in the manual. On average, ~90.50% of reads in individual libraries could be aligned to the reference pig genome using the STAR alignment tool (v 2.5.3a) (Supplementary Fig. S1d).

Kallisto (v 0.44.0)^99^ was used to quantify gene expression and obtain TPM values. Fold changes in gene transcription levels were estimated using edgeR^100^ (v 3.22.5) based on read counts. Differentially expressed genes (|log_2_FC | > 1, FDR < 0.05) between the HFD and controls were identified with edgeR (v 3.22.5). IGV (v 2.3.91)^101^ was used to visualize the location of different genes and the expression data of selected genomic regions.

### ATAC-seq library preparation and sequencing

ATAC-seq was performed as previously reported^102^. Chopped frozen liver tissue was resuspended in homogenization buffer, and grounded under a homogeneous solution, followed by filtered with a cell strainer. Cell pellets were obtained by centrifugation. After iodixanol density gradient centrifugation, the nuclei band was collected from the resuspended sediment. Then 50,000 nuclei were resuspended in the Tn5 transposase reaction mix with two adapters. The transposition reaction was incubated at 37 °C for 30 min. The fragmented DNA was purified and amplified with a limited PCR cycle using index primers. After the PCR reaction, libraries were purified with the AMPure beads and library quality was assessed with Qubit. The clustering of the index-coded samples was performed on a cBot Cluster Generation System using TruSeq PE Cluster Kit v3-cBot-HS (Illumina) according to the manufacturer’s instructions. The libraries were sequenced using the Illumina Novaseq6000 PE150 platform by Novogene (Beijing, China).

### ATAC-seq data analysis

Quality control of raw sequencing data was performed using trim-galore (v 0.6.4, https://www.bioinformaticsbabrahamacuk/projects/trim_galore/) with the options of “-q 25 -phred33 -length 74 -e 0.1 -stringency 4 -paired”. High-quality reads were aligned to the reference pig genome (Sscrofa 11.1) using Bowtie2^103^ (v 2.2.6) with default settings. Mitochondrial alignments, low-quality alignments (*q* < 10) and PCR duplicates were removed using SAMtools^104^ (v 1.3.1) (Supplementary Fig. S8a). ATAC peaks were called using MACS2 (https://github.com/macs3-project/MACS)^105^ with the options of “--nomodel --extsize 200 --shift −100 --nomodel -B --SPMR --format=BEDPE --keep-dup=1 --qvalue=0.05”. We also checked the enrichment of ATAC peaks on transcription start site (TSS) regions and the correlation between ATAC peaks and compartment status. To obtain differentially accessible regions (DARs), we merged peaks from all samples to acquire a non-redundant peak set using bedtools2^106^ (v 2.27.1). Read pair numbers for each non-redundant peak were calculated using HTseq (v 0.8.0) with the options of “--format=bam --order=pos --stranded=no --nonunique=all”. We detected potential DARs (|log_2_FC| > 2 and FDR < 0.001) using EdegR (v 3.22.5) based on read pair counts. Motif enrichment analysis was performed using the AME (Analysis of Motif Enrichment) tool packed in the MEME suite^107^ (v 5.3.3) with default settings based on JASPAR database^108^.

### ChIP-seq assays

To measure the activities of putative enhancers and promoters involved in PEIs, we performed ChIP-seq using antibodies against H3K27ac (a canonical histone mark of enhancers) and H3K4me3 (an active histone mark of promoters) for two biological replicates for each of the six developmental stages and the HFD treatment. The ChIP-seq experiments were performed as previously described^109^. Chromatin was prepared from formaldehyde-fixed liver tissues and fragmented with a sonicator to an average fragment size of 200–500 bp. Half of the soluble chromatin was stored at –20 °C for DNA sequencing (input control) and the remaining used for immunoprecipitation reacting with H3K27ac (ab4729, Abcam) or H3K4me3 (9751, CST) antibodies. For both input sequencing DNA and immunoprecipitated DNA, each ChIP-seq library was sequenced on an Illumina HiSeq X Ten platform to generate 150 bp paired-end reads.

### ChIP-seq data processing

High-quality ChIP-seq data were mapped to the reference pig genome (Sscrofa 11.1) using BWA (v 0.7.15) (Supplementary Fig. S1e), allowing up to two mismatches. SAMtools (v 1.3.1)^104^ was employed to remove potential PCR duplicates. We merged the bam files of biological replicates. H3K27ac and H3K4me3 peaks were called using SICER (v 1.1)^110^ with a cutoff of FDR < 0.05 for each sample and the merged samples. The peaks occurring in both the merged sample and at least one biological replicate were retained for subsequent analyses. The strength of H3K27ac or H3K4me3 signal was measured as log_2_(mark FPKM / input FPKM). The active promoter was defined as the 5 kb promoter bin overlaps with H3K4me3 peak in the length of at least 1 bp. Super-enhancer (SE) peaks were identified using the standard Rank Ordering of Super-Enhancers (ROSE) algorithm^111^. Briefly, neighboring enhancer elements (within 12.5 kb) identified using H3K27ac peaks were merged and ranked using the H3K27ac signal to identify a tangent with a slope of 1. The enhancers above the tangent were then defined as super-enhancer (SE) peaks, while those below the tangent were classified as regular-enhancer (RE) peaks. Genomic regions contacting distal promoters were identified as poised-enhancers (PEs) when not overlapping with a H3K27ac peak.

### Time-series analysis of chromatin architectures and gene expression

MaSigPro (v 3.12)^49^ incorporated with linear models (GLMs) was utilized to identify temporally dynamic compartment and TAD profiles. For switched compartments between neighboring developmental stages and cTADs across developmental stages, A-B indexes and *D*-scores were, respectively, employed as inputs for MaSigPro, and the change in values over the time series was selected when the goodness-of-fit (*R*^2^) was 0.6. *K*-means clustering was then performed to select the optimal number of clusters.

Short Time-series Expression Miner (STEM, v 1.3.13)^65^, an algorithm specifically designed for clustering short time-series data, was also used to identify the predominant trends of dynamic RPS profiles for eight a priori gene sets representing core liver functions during development (FDR-corrected *P* < 0.05, multiple hypothesis test).

### Acquisition of gene sets

Gene sets related to specific liver functions or NAFLD were obtained from Gene Ontology (GO), KEGG, Reactome, or related studies. All human genes were converted to their respective orthologs in the reference pig genome. The eight a priori gene sets linked to core liver functions included hematopoiesis (*n* = 719, GO: 0030097), amino acid metabolism (*n* = 308, GO: 0006520), fatty acid metabolism (*n* = 740, GO: 0008610, GO: 0006635, and R-HSA-1989781), glucose metabolism (*n* = 97, GO: 0061621 and GO: 0006094), tricarboxylic acid cycle (*n* = 41, GO: 0006099), bile acid metabolism (*n* = 30, obtained from^112,113^), drug metabolism (*n* = 130, retrieved from^114–116^), and hepatopathy-related immunity (*n* = 66, obtained from^117–121^). The 126 genes associated with NAFLD were converted from 127 human genes in the KEGG pathway ko04932. Signature genes for human HCC of S-I, -II, -III subtypes were obtained from^23^ (S-I: *n* = 43, S-II: *n* = 23, S-III: *n* = 410), and were converted to pig orthologs (S-I: *n* = 32; S-II: *n* = 15; S-III: *n* = 263) for further analysis.

### Functional enrichment analysis

Function enrichment analyses were performed using Metascape (http://metascape.org)^50^ with default parameters. Genes in the pig genome were converted to human orthologs, which were used as inputs for the enrichment. Human (*Homo sapiens*) was chosen as the target species, and enrichment analysis was performed against all genes in the genome as the background set, with Gene Ontology-biological processes (GO-BP) as the ontology test set. The top ten most statistically significant terms were selected as outputs.

### Trait-associated SNP enrichment analysis

We downloaded 146,690 unique human trait-associated SNPs from the NHGRI-EBI GWAS Catalog (https://www.ebi.ac.uk/gwas/, June 1, 2021)^122^. These SNPs were assigned to 77,917 loci in the reference pig genome (Sscrofa 11.1) using the UCSC LiftOver tool (https://genome.ucsc.edu/cgi-bin/hgLiftOver). Of these, 73,363 noncoding SNPs (or 94.16%) linked to 4514 traits or diseases were used for subsequent analyses. To quantify the degree of association between certain genomic regions and traits or diseases, we calculated the enrichment score (i.e., relative density) of noncoding SNPs for different genomic regions^86^. The significance of the enrichment score was calculated using a *χ*^2^ test for each trait or disease that contained more than 50 SNPs.

## Supplementary information

Supplementary figures and tables

Supplementary data S3

Supplementary data S1

Supplementary data S2

## Data Availability

The Hi-C, RNA-seq, and ChIP-seq data generated in this study are available at Sequence Read Archive (SRA) under the BioProject PRJNA721459 and Gene Expression Omnibus (GEO) under the accession number GSE176387.
