# Supplementary material for: Dynamic 3D genome reorganization during development and metabolic stress of the porcine liver
Source: Cell Discov. 2022 Jun 14;8:56. doi: 10.1038/s41421-022-00416-z (PMC9197842; doi:10.1038/s41421-022-00416-z)
Supplement: Supplementary file 1 — Supplementary figures and tables [file 41421_2022_416_MOESM1_ESM.pdf]

Supplementary Information

Table of Contents

Supplementary Figures S1–16

Fig. S1 Data summary of Hi-C, RNA-seq and ChIP-seq.

Fig. S2 Multi-scale measurement of chromatin architecture and gene expression similarities between samples during development and HFD feeding.

Fig. S3 Form-function dynamics in porcine liver genomes during development and under HFD treatment.

Fig. S4 Comparison of inter-chromosomal spatial distances and nuclear radius during liver development.

Fig. S5 Genomic features of compartments and TADs.

Fig. S6 Dynamic compartmentalization and reorganization of TADs affect gene expression.

Fig. S7 Examples of genes located in compartment-switching regions and changing TADs.

Fig. S8 Chromatin accessibility between prenatal (E80) and adult (2Y) stages.

Fig. S9 Rewiring of PEIs dynamically regulates gene expression in a developmental-dependent manner.

Fig. S10 Genes with the developmental-dependent changes in RPS are related to functional transitions during liver development.

Fig. S11 STEM clustering of RPS profiles across developmental stages for eight a priori gene sets related to core liver functions at prenatal (hematopoiesis) and postnatal stages (metabolism of amino acid, fatty acid, glucose, bile acid, and drug; tricarboxylic acid cycle; and immunity).

Fig. S12 Examples of PEI rewiring related to functional transitions during liver development.

Fig. S13 Gene expression changes between HFD- and normal diet-fed pigs.

Fig. S14 Promoter-promoter interaction profiles responding to liver metabolic stress in pigs.

Fig. S15 The loose chromatin architecture allows for the transcription of extensive genomic regions during early liver development.

Fig. S16 Human trait-associated noncoding SNPs were enriched in enhancers in the porcine liver.

Supplementary Tables S1–2

Table S1 Functional description of the genes mentioned in this study.

Table S2 Information of primers and genomic locations for target gene promoters and enhancers validated using the Dual-Luciferase reporter assay.

Supplementary Figures

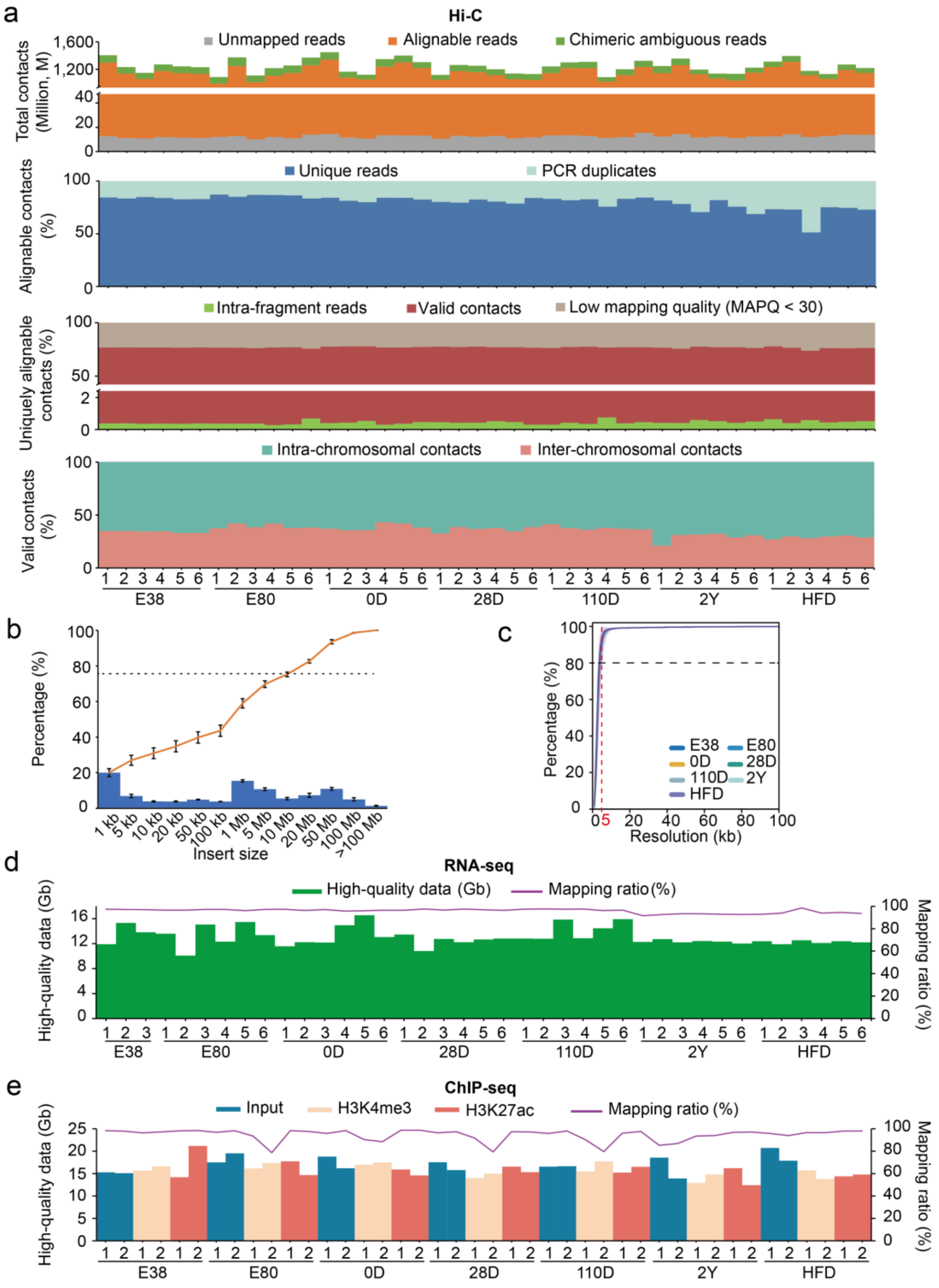

Supplementary Fig. S1 Data summary of Hi-C, RNA-seq and ChIP-seq. We collected a total of 42 liver samples from seven groups of pigs (including six developmental stages and a high fat diet-feeding group), which have six biological replicates for each stage/group, except the stage of embryonic day 38 (E38), which was pooled from livers of 14 embryos, and further been equally divided into six parts. **a** Data summary of *in situ* Hi-C (n = 42, six replicates for each stage/group). **b** Insert

size distribution of intra-chromosomal contacts of *in situ* Hi-C data. The column represents the proportion of paired contacts within each interval distance. The curve represents the cumulative percentage. Data are represented as mean ± SD. **c** Resolutions of intra-chromosomal maps constructed using the merged Hi-C data from six replicates of each developmental stage and the HFD treatment. Map resolution is defined as the smallest bin size where 80% of the bins contain at least 1,000 reads in order to allow reliable discerning of local features[89]. Data are shown as mean ± SD. **d** RNA-seq data summary (n = 39, six replicates for each stage/group, except the stage of E38 which has three replicates). **e** ChIP-seq data summary (n = 42, two replicates for each stage/group for input control, H3K27ac, and H3K4me3).

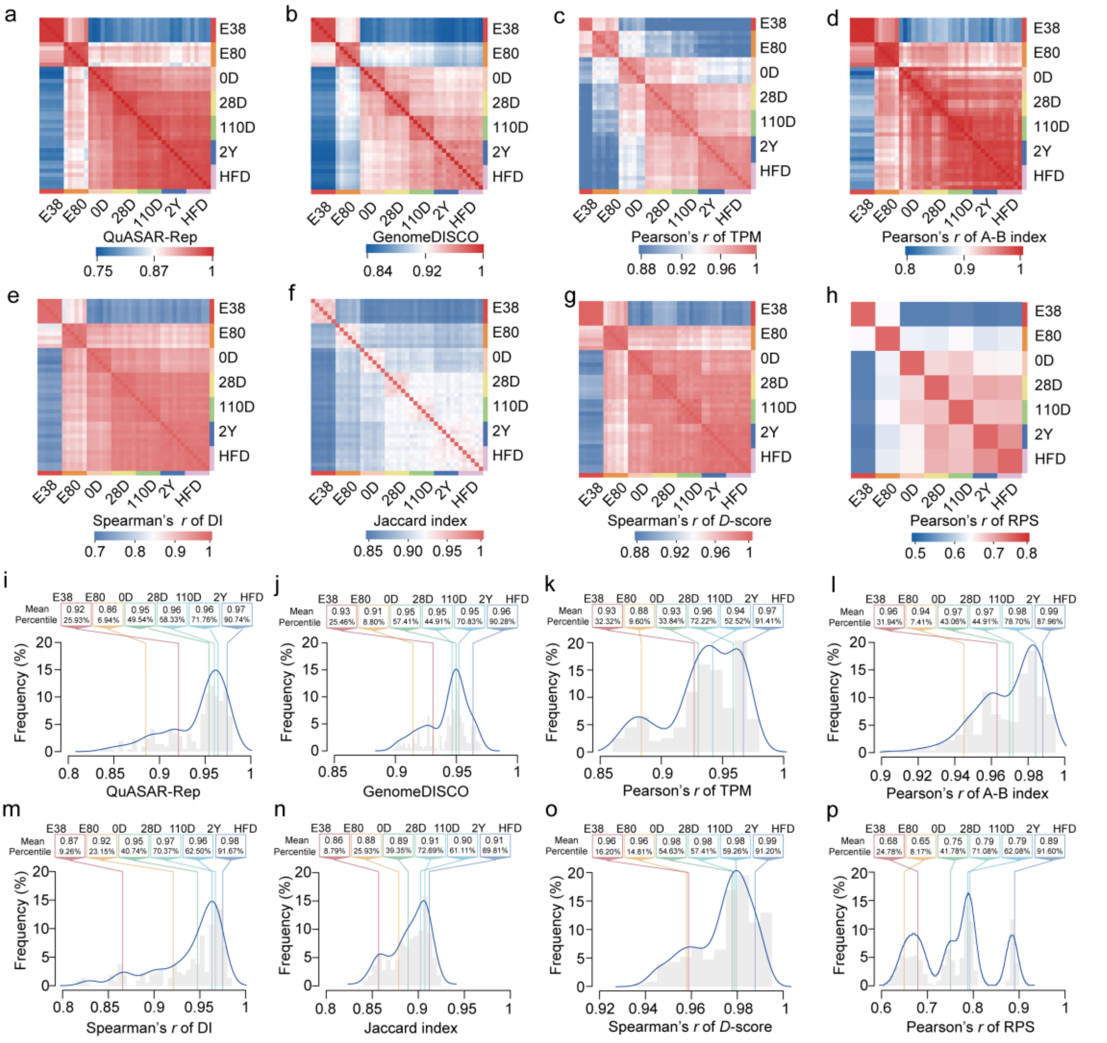

Supplementary Fig. S2 Multi-scale measurement of chromatin architecture and gene expression similarities between samples during development and HFD feeding. a–h, Correlations of chromatin architecture were evaluated by QuASAR-Rep (**a**) and GenomeDISCO (**b**) for the Hi-C maps; A-B index (**d**) and Directionality Index (DI, **e**) for 20-kb genomic bins; Jaccard index (**f**) for TADs, *D*-score (**g**) for cTADs; and regulatory potential score (RPS, **h**) for genes. The correlations of gene expression were determined using the RNA-seq data (**c**). **i–p,** Distribution of similarities between samples of consecutive stages, as well as between stage 2Y and an HFD-feeding group. To generate the frequency distribution of similarity for RPS obtained using the combined Hi-C data from six replicates within each stage/group, we performed bootstrapping by randomly resampling the RPS of 10^4 genes from a pool of 16,435 genes (random number = 10^3). The vertical lines correspond to the percentiles of similarities for comparisons between stages/groups, indicating the empirical probabilities of the observed values.

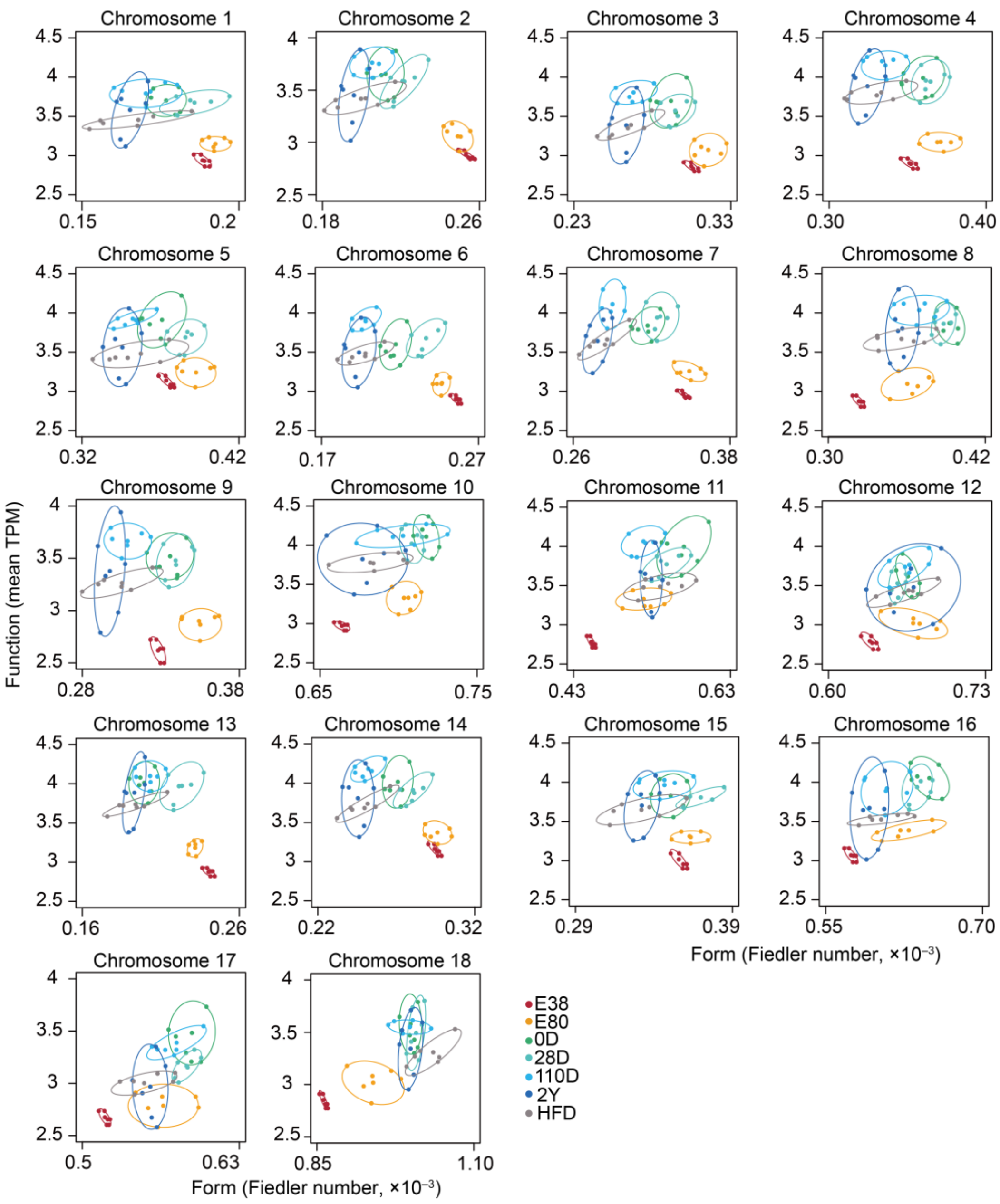

Supplementary Fig. S3 Form-function dynamics in porcine liver genomes during development and under HFD treatment. Chromosomal form was characterized by the network connectivity (Fiedler number, FN) of chromatin contacts based on Hi-C data. Genomic function was inferred by the expression level (mean TPM) based on RNA-seq data, as described in the **Materials and methods**. For each chromosome, form-function portraits of six technical (E38) or six biological replicates (other five stages and the HFD-fed pigs) are circled by the minimum volume ellipse. Note the latter four postnatal stages exhibited more similarity in form-function features than the two prenatal stages.

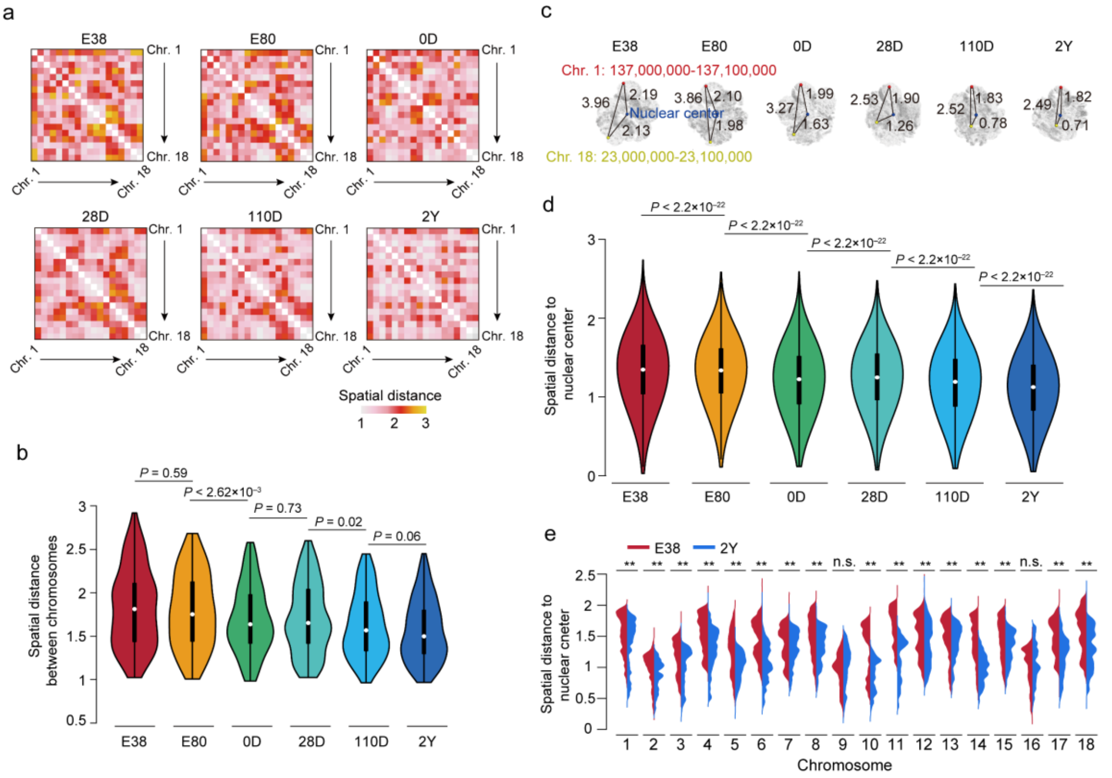

Supplementary Fig. S4 Comparison of inter-chromosomal spatial distances and nuclear radius during liver development. The 3D genome structure of porcine livers across six developmental stages were separately reconstructed using intra- (20-kb resolution) and inter-chromosomal (1-Mb resolution) interactions with the Python package miniMDS[96]. **a**, **b** Comparison of inter-chromosomal spatial distances during development. **c** Visualization of 3D genome structure using PyMOL (v 2.5.2, https://pymol.org/2/). Two 100-kb regions in chromosomes 1 and 18 are marked; their respective distances to the nuclear center and each other gradually decreased during development. **d** Comparison of the nuclear radius (reflected by the average distance to the nuclear center of mass) during development. **e** Comparison of the nuclear radius between prenatal E38 and adult 2Y for each autosome. *P*-values in **b**, **d**, and **e** were calculated using the Wilcoxon rank-sum test. n.s., $P \geq 0.05$; $^{**}P < 0.01$.

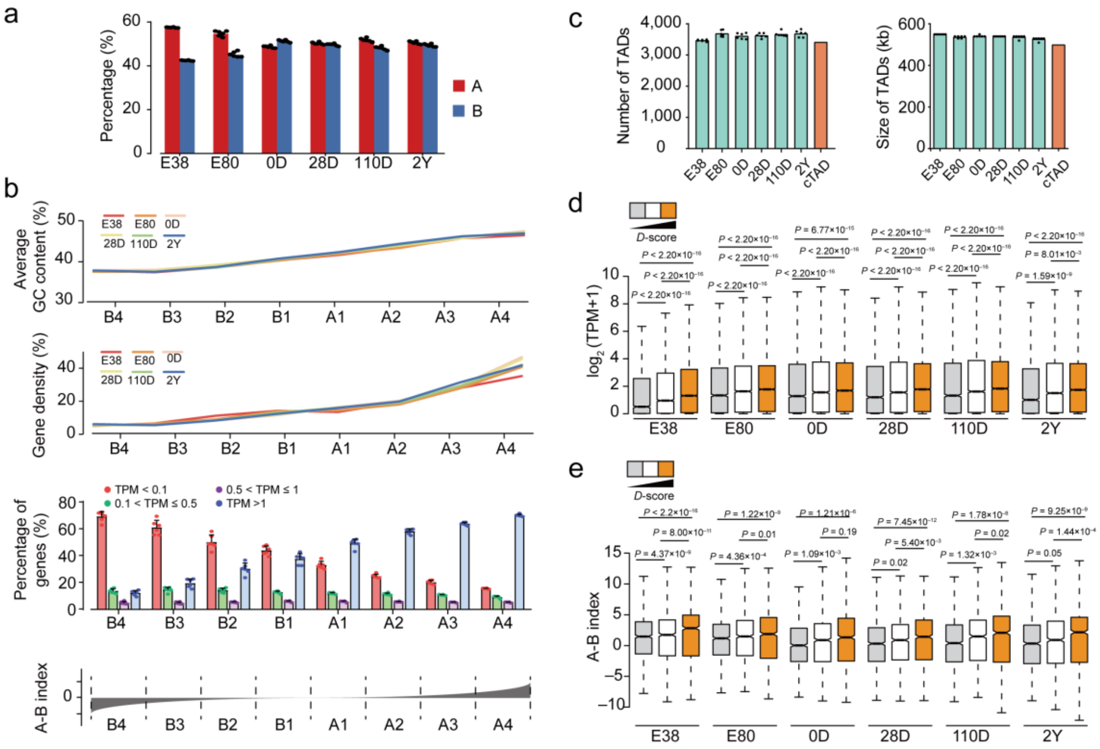

Supplementary Fig. S5 Genomic features of compartments and TADs. a Length proportions of A/B compartments. The length ratio of A compartments to B compartments was estimated at around 6:4 before birth, declining to 5:5 after birth. The different dots represent the six replicates for each developmental stage. **b** According to the A-B index, A (A1–4) and B compartments (B1–4) were divided into four equal parts across the different stages. GC content (top), gene density (middle), and proportion of genes with different expression levels (bottom) are displayed. The dots in the histogram denote the different replicates. **c** Numbers and sizes of TADs and consensus TADs (cTADs). The definition of cTAD can be found in the **Materials and methods.** The dots represent replicates at each stage. **d** Gene expression and **e** A-B index of genes within TADs of low, median and high *D*-score (three percentiles). *P*-values in **d** and **e** were calculated using the Wilcoxon rank-sum test.

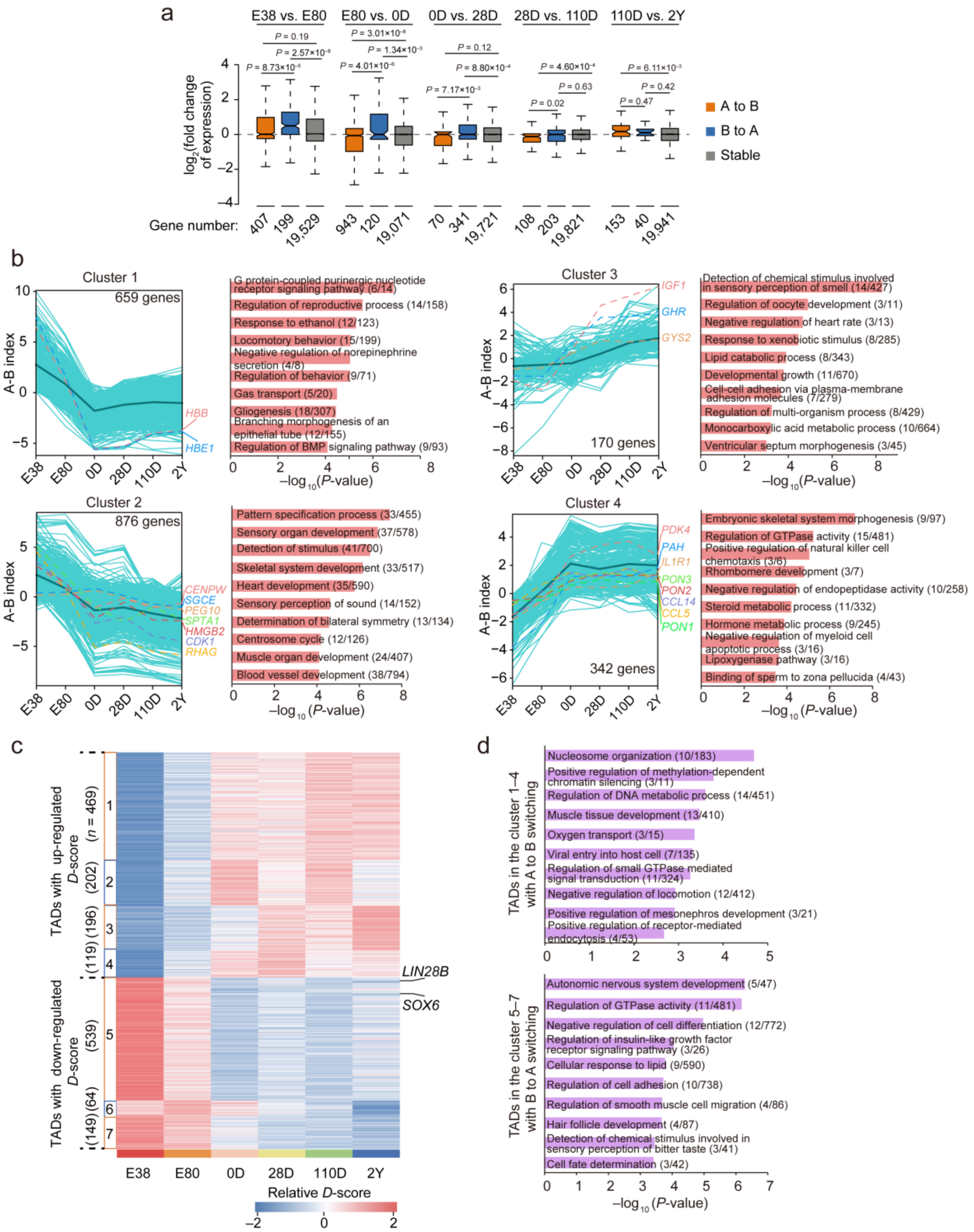

Supplementary Fig. S6 Dynamic compartmentalization and reorganization of TADs affect gene expression. a Expression changes in genes that are located in compartment-switching regions between consecutive stages. *P*-values were calculated using the Wilcoxon rank-sum test. **b** Clustering of genes with dynamic A-B index. Left: four representative gene clusters were detected using the maSigPro-GLM algorithm[49] (v 3.12). Right: functional enrichment of genes in each cluster. **c** Seven representative clusters of genes located in TADs with different *D*-scores during

development as estimated by the maSigPro-GLM algorithm[49]. **d** Functional enrichment of genes located in the changing TADs (inferred by *D*-scores) with switched compartments. Representative functional genes are labelled on the plot in **b** and **c**. Functional enrichment analyses in **b** and **d** were conducted using Metascape[50]. Only the top ten significantly enriched GO terms are showed.

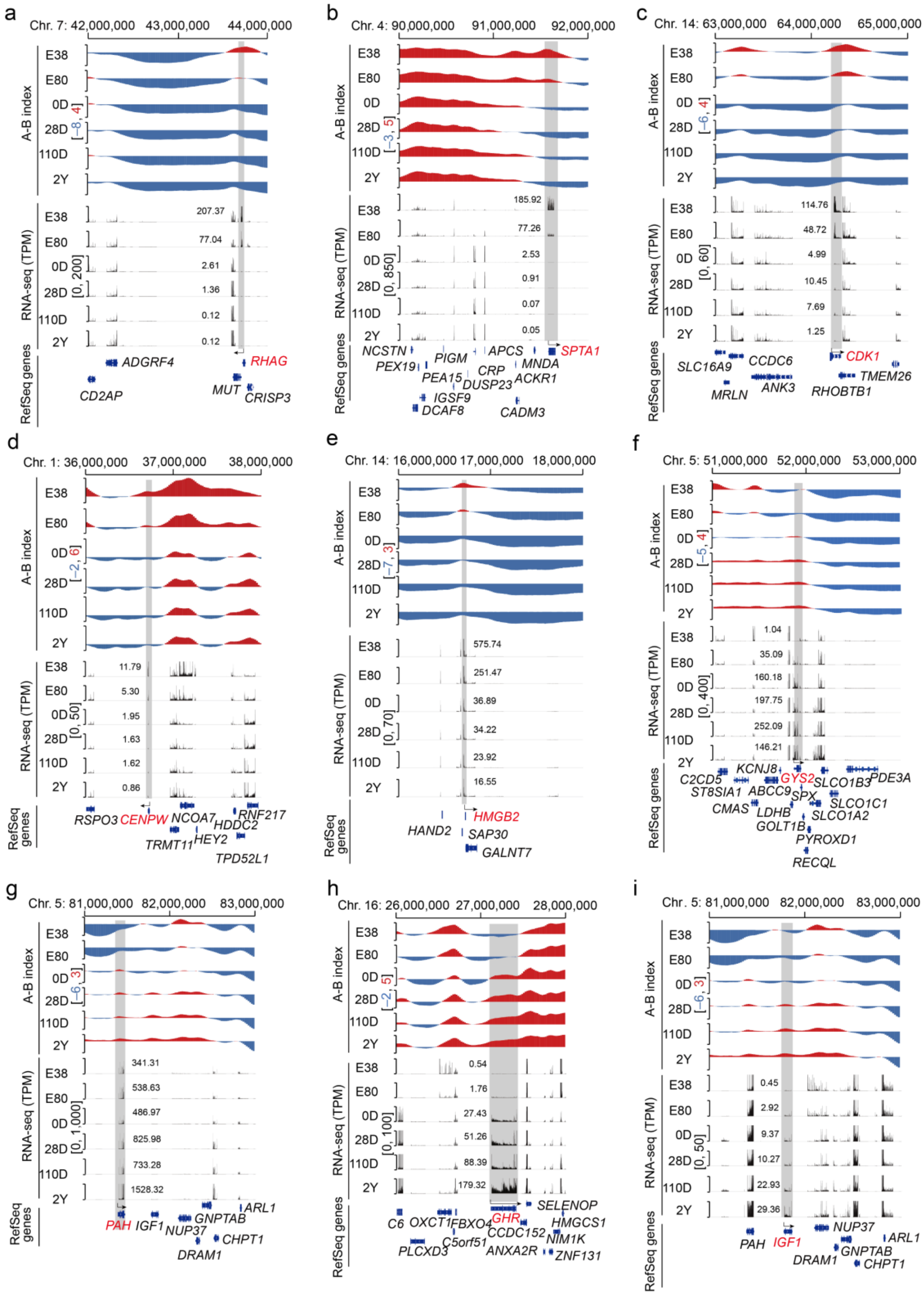
a
Chr. 7: 42,000,000 43,000,000 44,000,000
A-B index [−8, 4]
E38
E80
0D
28D
110D
2Y
RNA-seq (TPM) [0, 200]
207.37
77.04
2.61
1.36
0.12
0.12
RefSeq genes
ADGRF4
RHAG
CD2AP
MUT
CRISP3
b
Chr. 4: 90,000,000 91,000,000 92,000,000
A-B index [−3, 5]
RNA-seq (TPM) [0, 850]
185.92
77.26
2.53
0.91
0.07
0.05
NCSTN
PEX19
PIGM
PEA15
IGSF9
DCAF8
APCS
CRP
DUSP23
MNDA
ACKR1
CADM3
SPTA1
c
Chr. 14: 63,000,000 64,000,000 65,000,000
A-B index [−6, 4]
RNA-seq (TPM) [0, 60]
114.76
48.72
4.99
10.45
7.69
1.25
SLC16A9
CCDC6
MRLN
ANK3
CDK1
RHOBTB1
TMEM26
d
Chr. 1: 36,000,000 37,000,000 38,000,000
A-B index [−2, 6]
RNA-seq (TPM) [0, 50]
11.79
5.30
1.95
1.63
1.62
0.86
RSPO3
CENPW
NCOA7
TRMT11
HEY2
HDDC2
RNF217
TPD52L1
e
Chr. 14: 16,000,000 17,000,000 18,000,000
A-B index [−7, 3]
RNA-seq (TPM) [0, 70]
575.74
251.47
36.89
34.22
23.92
16.55
HAND2
HMGB2
SAP30
GALNT7
f
Chr. 5: 51,000,000 52,000,000 53,000,000
A-B index [−5, 4]
RNA-seq (TPM) [0, 400]
1.04
35.09
160.18
197.75
252.09
146.21
C2CD5
ST8SIA1
CMAS
KCNJ8
ABCC9
LDHB
GOLT1B
GYS2
SPX
PYROXD1
RECQL
SLCO1B3
SLCO1C1
SLCO1A2
PDE3A
g
Chr. 5: 81,000,000 82,000,000 83,000,000
A-B index [−6, 3]
RNA-seq (TPM) [0, 1,000]
341.31
538.63
486.97
825.98
733.28
1528.32
PAH
IGF1
NUP37
DRAM1
GNPTAB
CHPT1
ARL1
h
Chr. 16: 26,000,000 27,000,000 28,000,000
A-B index [−2, 5]
RNA-seq (TPM) [0, 100]
0.54
1.76
27.43
51.26
88.39
179.32
C6
PLCXD3
OXCT1
FBXO4
C5orf51
GHR
CCDC152
ANXA2R
SELENOP
HMGCS1
NIM1K
ZNF131
i
Chr. 5: 81,000,000 82,000,000 83,000,000
A-B index [−6, 3]
RNA-seq (TPM) [0, 50]
0.45
2.92
9.37
10.27
22.93
29.36
PAH
IGF1
NUP37
DRAM1
GNPTAB
CHPT1
ARL1

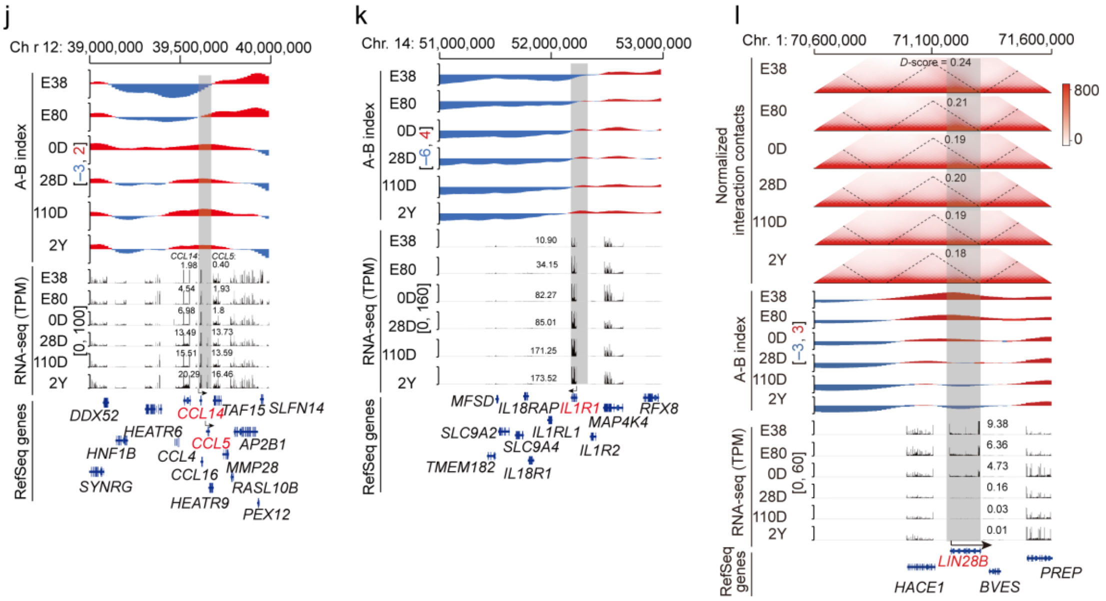

Supplementary Fig. S7 Examples of genes located in compartment-switching regions and changing TADs. a–k Dynamic compartment status and corresponding gene expression for **(a)** *RHAG*, **(b)** *SPTA1*, **(c)** *CDK1*, **(d)** *CENPW*, **(e)** *HMGB2*, **(f)** *GYS2*, **(g)** *PAH*, **(h)** *GHR*, **(i)** *IGF1*, **(j)** *CCL5* and *CCL14*, and **(k)** *IL1R1*. **l** Representative TADs (containing *LIN28B*) with the reduced *D*-score and A to B compartment switching during development.

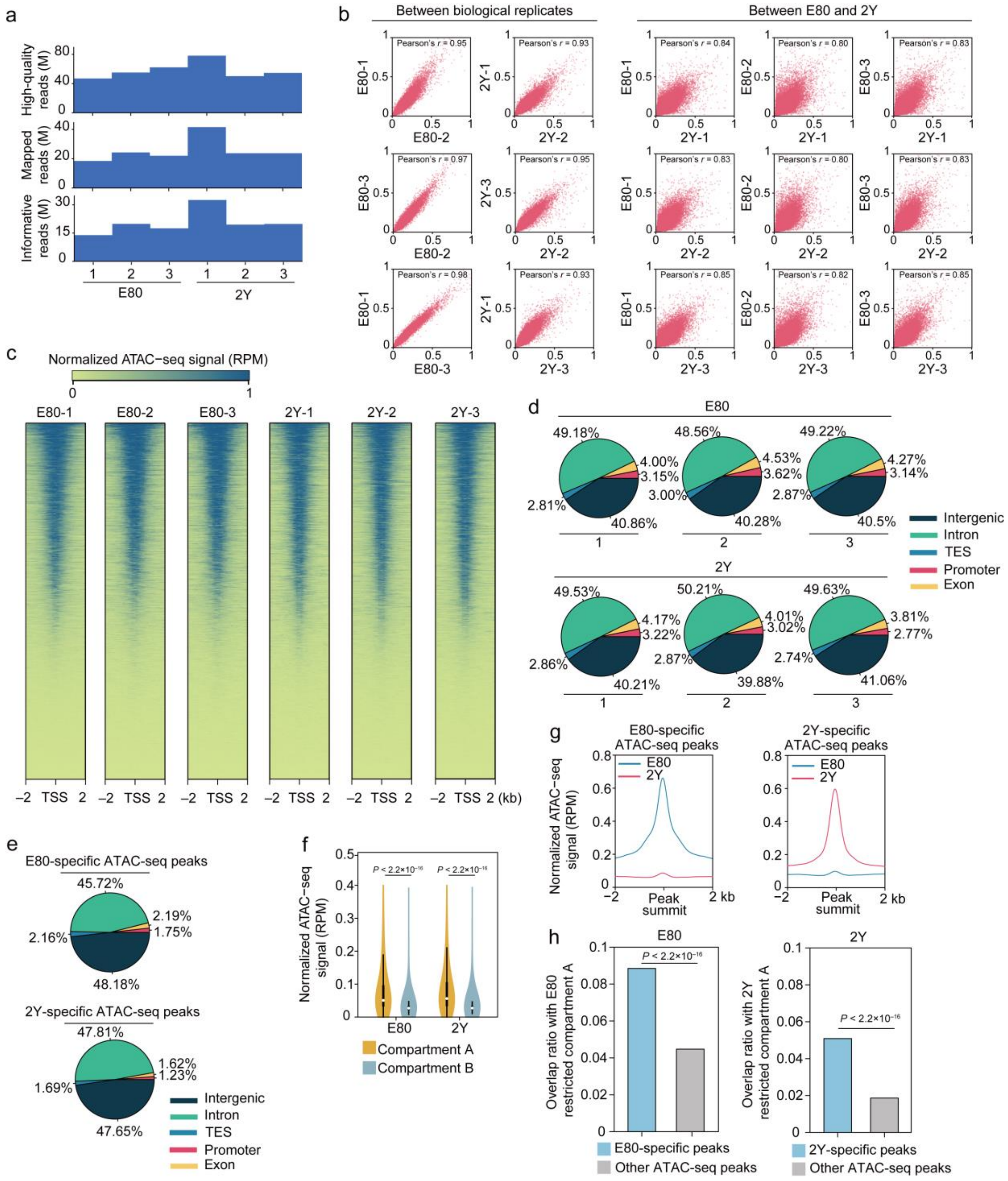
a
High-quality reads (M)
Mapped reads (M)
Informative reads (M)
E80
2Y
b
Between biological replicates
Between E80 and 2Y
Pearson's r = 0.95
Pearson's r = 0.93
Pearson's r = 0.84
Pearson's r = 0.80
Pearson's r = 0.83
Pearson's r = 0.97
Pearson's r = 0.95
Pearson's r = 0.83
Pearson's r = 0.80
Pearson's r = 0.83
Pearson's r = 0.98
Pearson's r = 0.93
Pearson's r = 0.85
Pearson's r = 0.82
Pearson's r = 0.85
c
Normalized ATAC−seq signal (RPM)
E80-1
E80-2
E80-3
2Y-1
2Y-2
2Y-3
−2 TSS 2 (kb)
d
E80
2Y
Intergenic
Intron
TES
Promoter
Exon
e
E80-specific ATAC-seq peaks
2Y-specific ATAC-seq peaks
f
Normalized ATAC−seq signal (RPM)
P < 2.2×10−16
Compartment A
Compartment B
g
E80-specific ATAC-seq peaks
2Y-specific ATAC-seq peaks
Normalized ATAC−seq signal (RPM)
Peak summit
h
Overlap ratio with E80 restricted compartment A
Overlap ratio with 2Y restricted compartment A
E80-specific peaks
Other ATAC-seq peaks
2Y-specific peaks

i

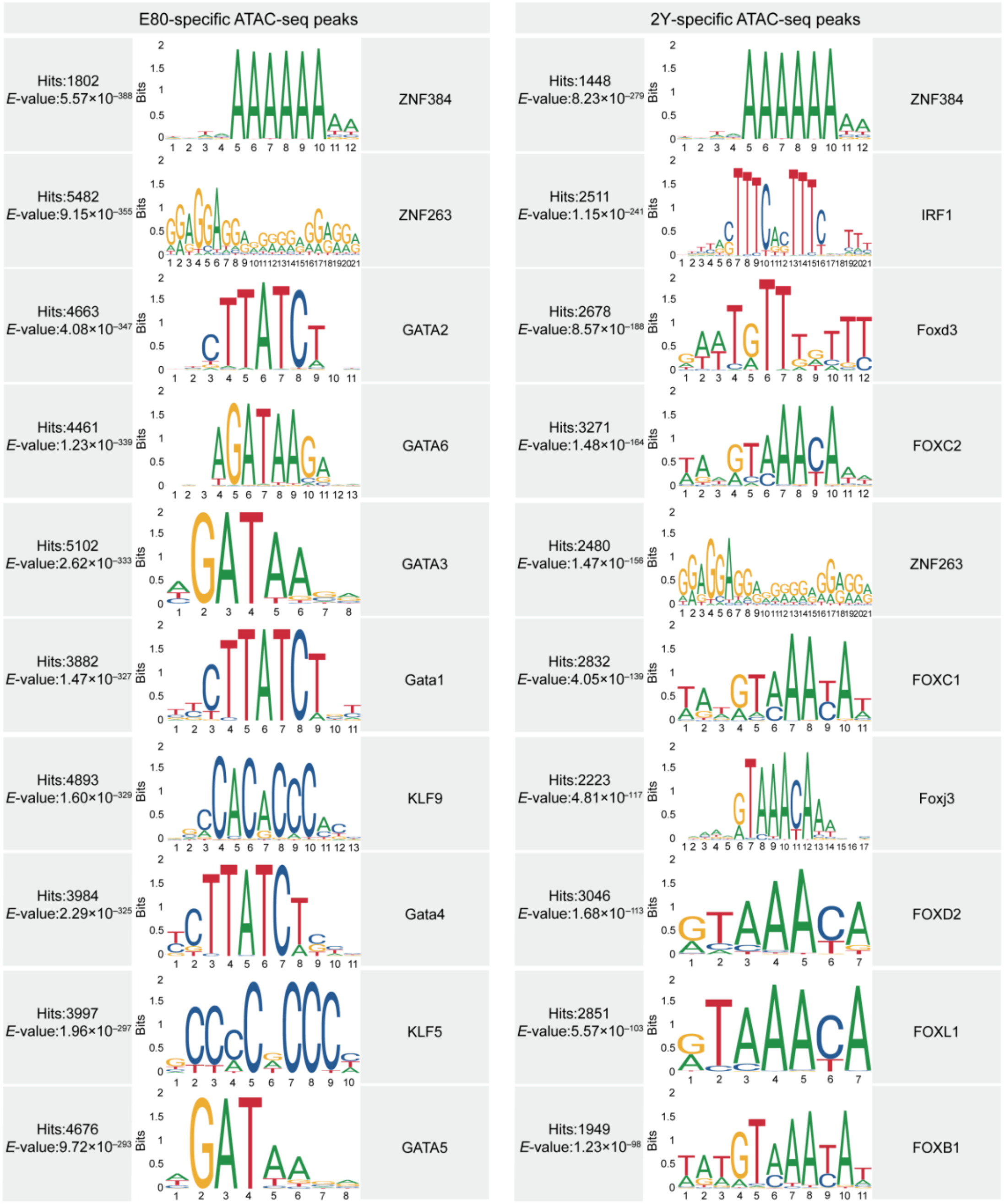

Supplementary Fig. S8 Chromatin accessibility between prenatal (E80) and adult (2Y) stages. a Data summary of ATAC-seq (n = 6, three replicates for two selected stages, *i.e.*, prenatal E80 and adult 2Y). **b** Scatterplots showing correlations of normalized ATAC-seq signals (reads per million, RPM) between samples. The genome-wide ATAC-seq signal was highly reproducible between biological replicates (average Pearson's r = 0.95), but were more dissimilar between stages (E80 and 2Y) (average Pearson's r = 0.83). **c** Heatmaps depicting the enrichment of normalized ATAC-seq signal cantered on transcription start site (TSS) and ordered by signal intensity. **d**, **e** Distribution of ATAC-seq peaks (**d**) and stage-specific ATAC-seq peaks (**e**) relative to genomic features. The peaks were classified into five distinct categories based on 1 bp overlap with features (i.e., promoter, exon, intron, transcription end site

[TES], and intergenic regions). **f** Normalized ATAC-seq signal in each 20 kb bins for compartment A (orange) or B (blue) regions. Statistical significance was calculated by the Wilcoxon rank-sum test. **g** Average of normalized ATAC-seq signal (RPM) in a 4 kb region cantered on stage-specific peaks between E80 and 2Y. **h** Overlap of stage-specific ATAC-seq peaks or rest peaks with stage-restricted compartment A regions. Statistical significance was determined by two-proportions test. **i** Top ten enriched transcription factor binding motifs within stage-specific ATAC-seq peaks, as identified by JASPAR database[108].

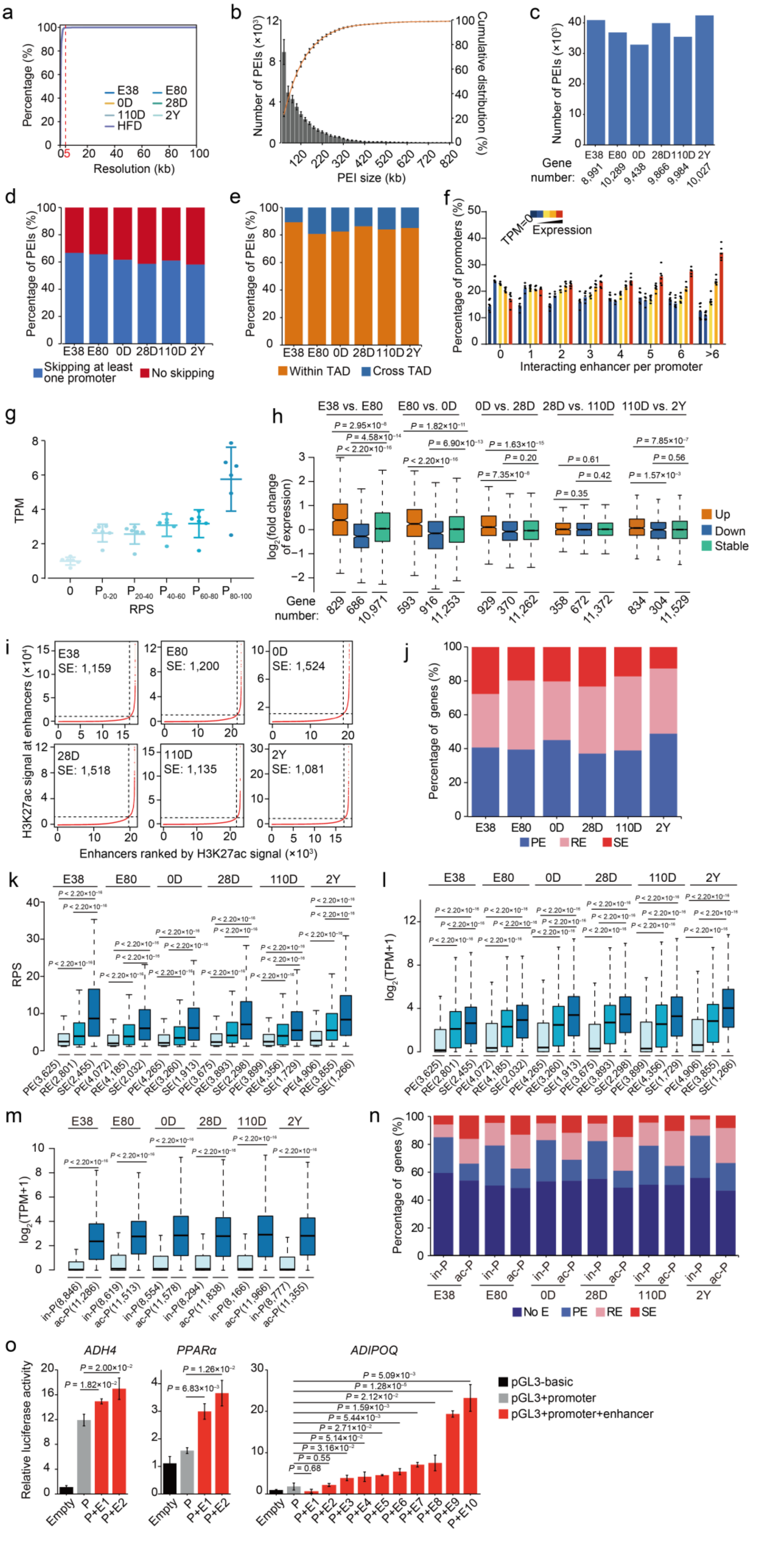

a
Percentage (%)
Resolution (kb)
E38
E80
0D
28D
110D
2Y
HFD
b
Number of PEIs (×10³)
Cumulative distribution (%)
PEI size (kb)
c
Number of PEIs (×10³)
Gene number:
E38 E80 0D 28D 110D 2Y
8,991 10,289 9,438 9,866 9,984 10,027
d
Percentage of PEIs (%)
Skipping at least one promoter
No skipping
e
Percentage of PEIs (%)
Within TAD
Cross TAD
f
Percentage of promoters (%)
TPM=0
Expression
Interacting enhancer per promoter
g
TPM
RPS
h
E38 vs. E80
E80 vs. 0D
0D vs. 28D
28D vs. 110D
110D vs. 2Y
log₂(fold change of expression)
Up
Down
Stable
Gene number:
829 686 10,971 593 916 11,253 929 370 11,262 358 672 11,372 834 304 11,529
i
H3K27ac signal at enhancers (×10⁴)
E38 SE: 1,159
E80 SE: 1,200
0D SE: 1,524
28D SE: 1,518
110D SE: 1,135
2Y SE: 1,081
Enhancers ranked by H3K27ac signal (×10³)
j
Percentage of genes (%)
PE
RE
SE
k
RPS
l
log₂(TPM+1)
m
log₂(TPM+1)
n
Percentage of genes (%)
in-P
ac-P
No E
PE
RE
SE
o
ADH4
PPARα
ADIPOQ
Relative luciferase activity
pGL3-basic
pGL3+promoter
pGL3+promoter+enhancer
Empty
P

Supplementary Fig. S9 Rewiring of PEIs dynamically regulates gene expression in a developmental-dependent manner. a Evaluation of resolutions for the intra-chromosomal Hi-C maps generated by the merged Hi-C data of six replicates at each stage and that of HFD-fed pigs. The curve represents the proportion of bins that contain at least 1,000 intra-chromosomal contacts in a relevant bin size. Map resolution is defined as the smallest bin size where 80% of the bins have at least 1,000 reads in order to allow reliable discerning of local features[89]. **b** Size distribution of PEIs identified at 5-kb resolution using the merged Hi-C data. PEIs larger than 20 kb were retained. The line represents the cumulative distribution. **c** Number of PEIs. **d** Proportion of enhancers interacting with the nearest promoter or skipping at least one promoter. **e** Proportion of PEIs within or across TADs. **f** Proportion of gene promoters from each expression category interacting with zero to more than six enhancers. **g** Gene expression was associated with RPS. Genes with RPS > 0 were divided equally into five different percentiles. Data are presented as mean ± SD. **h** Expression fold-changes for genes with differential RPS between consecutive stages. *P*-values were calculated using the Wilcoxon rank-sum test. **i** Determination of enhancer activity by analysing the distribution of H3K27ac signals using the ROSE algorithm[111]. **j** Proportion of genes interacting with super-enhancers (SEs), regular-enhancers (REs), and poised-enhancers (PEs). **k** RPS values and **l** expression levels of genes interacting with SEs, REs and PEs. **m** Expression levels of genes with active promoters (ac-P, enriched by H3K4me3 peaks) or inactive promoters (in-P). **n** Proportion of active or inactive promoters interacting with SEs, REs and PEs. **o** Validation of the enhancers identified in this study using a Dual-Luciferase Reporter Assay System in HEK-293T cells. Data shown are mean ± SD (n = 3). *P*-values were calculated using a two-sided Student's *t*-test.

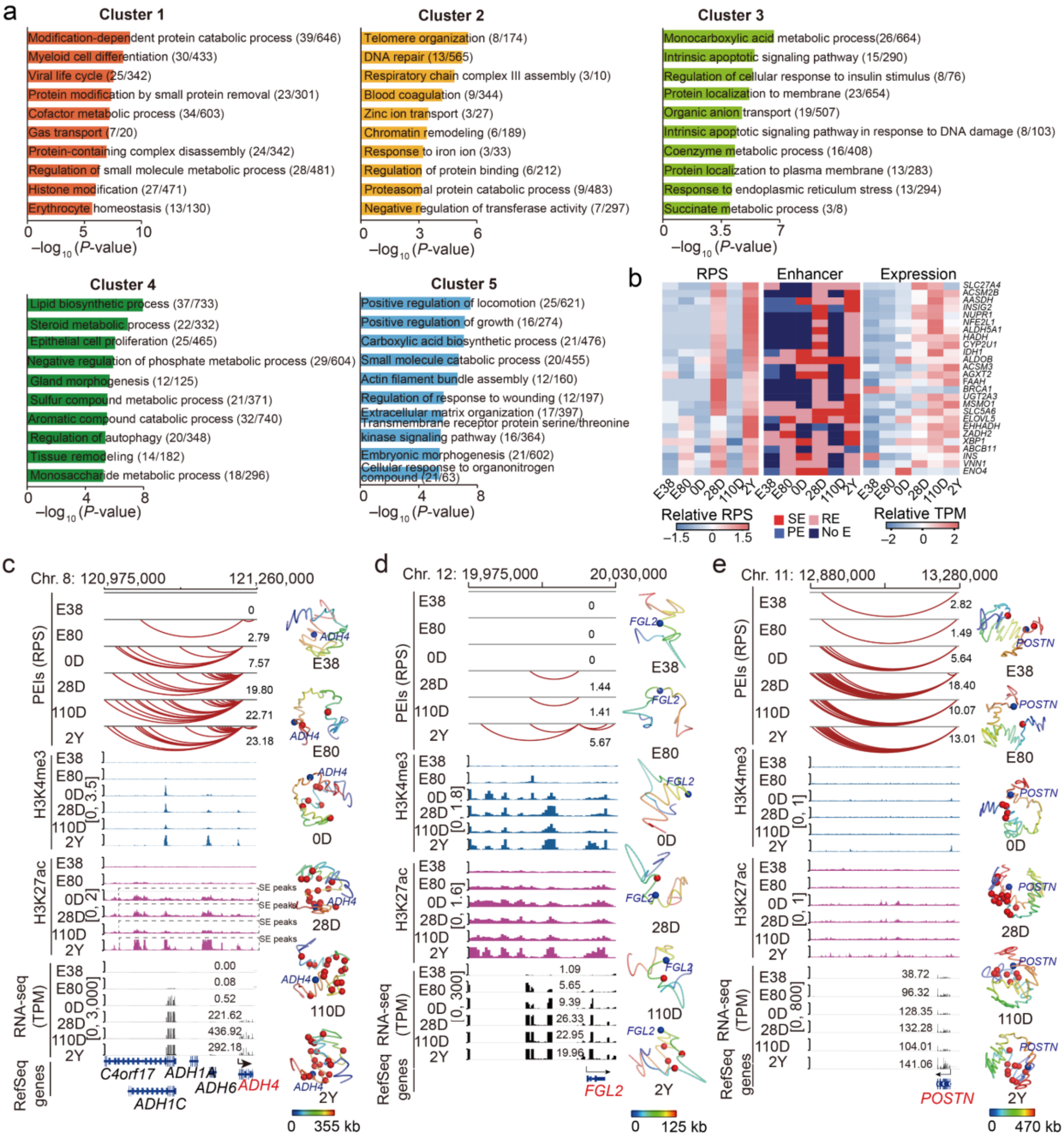

Supplementary Fig. S10 Genes with the developmental-dependent changes in RPS are related to functional transitions during liver development. a Top ten significantly enriched GO-BP terms (performed by the Metascape[50]) for five representative clusters of genes identified by the STEM algorithm based on dynamic RPS. **b** PEI rewiring of 26 genes that were enriched in the GO-BP term ‘monocarboxylic acid metabolic process’. The changes in RPS (left), interacting enhancers (middle) and gene expression (right) are shown. **c–e** Examples of PEI rewiring for HCC signature genes[23], *ADH4* (**c**), *FGL2* (**d**), and *POSTN* (**e**). *ADH4* is a signature gene for a less aggressive HCC subtype (S-I, good prognosis) and is involved in drug metabolism. *FGL2* and *POSTN* are signature genes for a more aggressive HCC subtype (S-III, poor prognosis) and participate in leukocyte activation. Left: Schematics of PEIs, H3K4me3 and H3K27ac signals, and transcription. Dashed boxes highlight SE peaks. Right: 3D structural models and Hi-C contact maps of the

corresponding genomic region (seven 5-kb bins up- and down-stream from the bin containing the TSS). Gene promoters (blue spheres) and enhancers (red spheres) are shown in the 3D models.

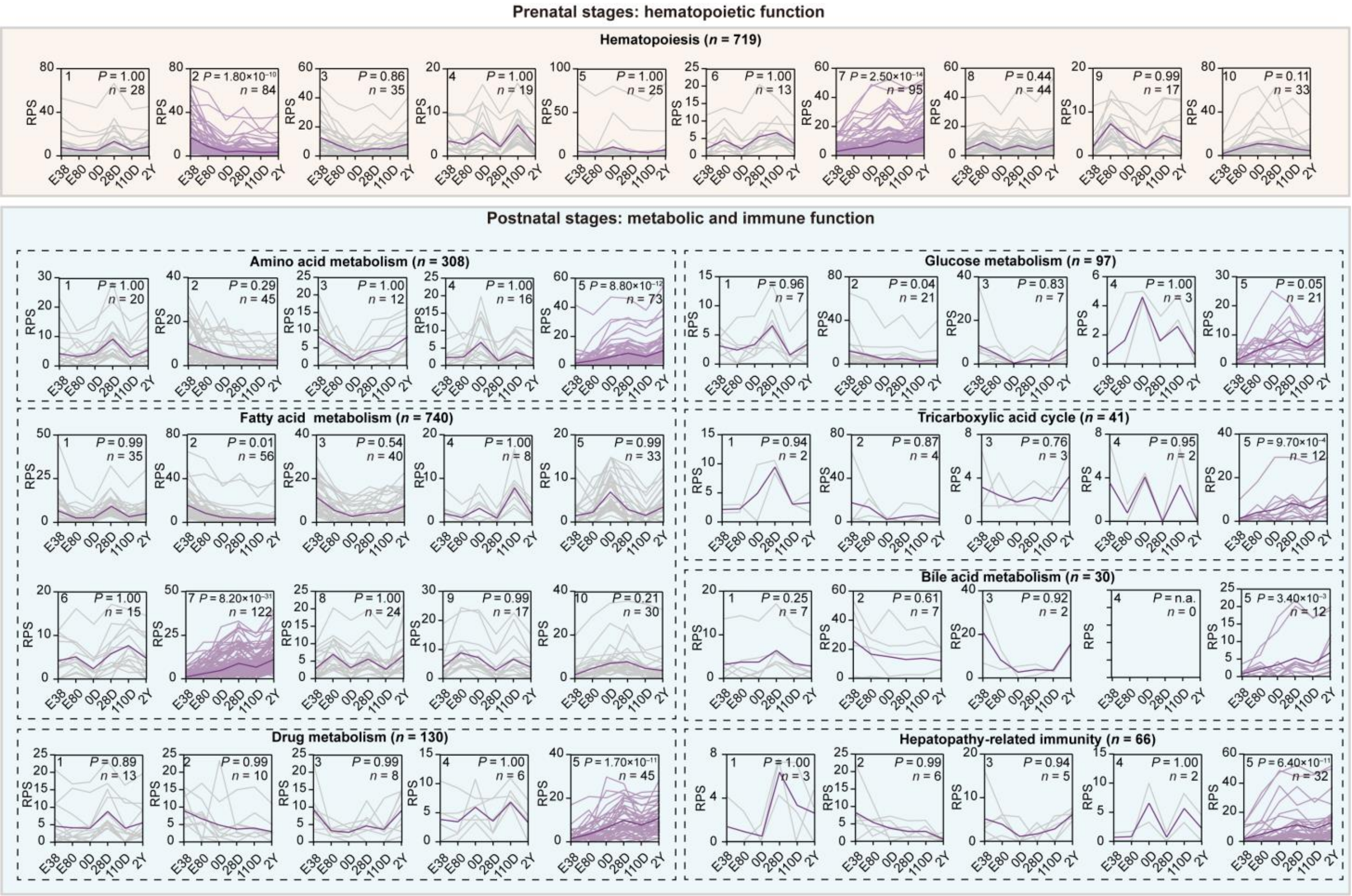

Supplementary Fig. S11 STEM clustering of RPS profiles across developmental stages for eight a priori gene sets related to core liver functions at prenatal (hematopoiesis) and postnatal stages (metabolism of amino acid, fatty acid, glucose, bile acid, and drug; tricarboxylic acid cycle; and immunity). Genes in each set were classified into five or ten profiles based on RPS changes during development. The bold purple lines in each plot represent the mean RPS, and the grey lines represent the RPS of genes in relevant cluster during development. FDR-corrected *P*-values were obtained from multiple hypothesis testing.

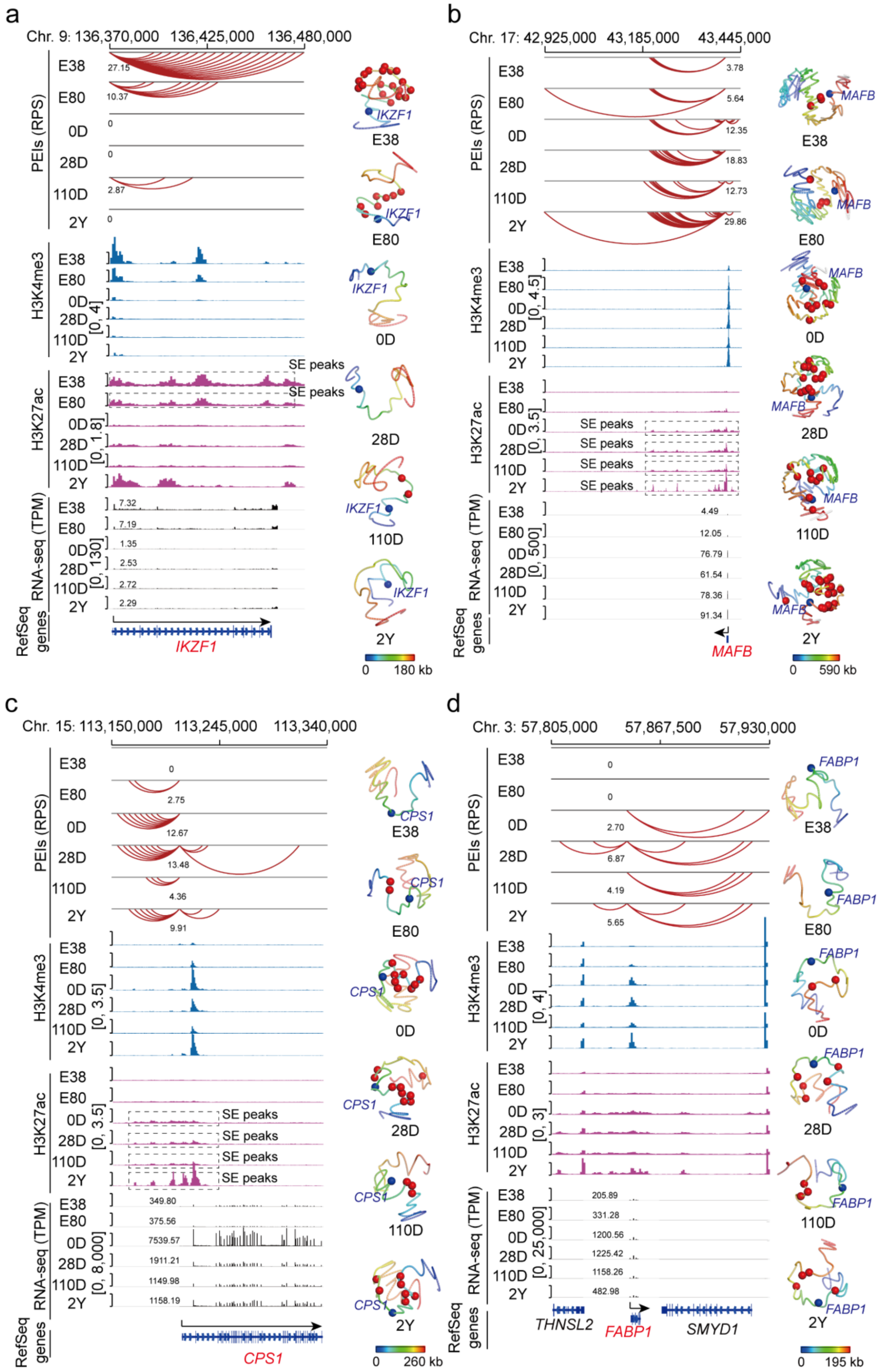
a
Chr. 9: 136,370,000 136,425,000 136,480,000
PEIs (RPS)
H3K4me3
H3K27ac
RNA-seq (TPM)
RefSeq genes
IKZF1
SE peaks
0 180 kb
b
Chr. 17: 42,925,000 43,185,000 43,445,000
MAFB
SE peaks
0 590 kb
c
Chr. 15: 113,150,000 113,245,000 113,340,000
CPS1
SE peaks
0 260 kb
d
Chr. 3: 57,805,000 57,867,500 57,930,000
THNSL2
FABP1
SMYD1
0 195 kb
E38
E80
0D
28D
110D
2Y

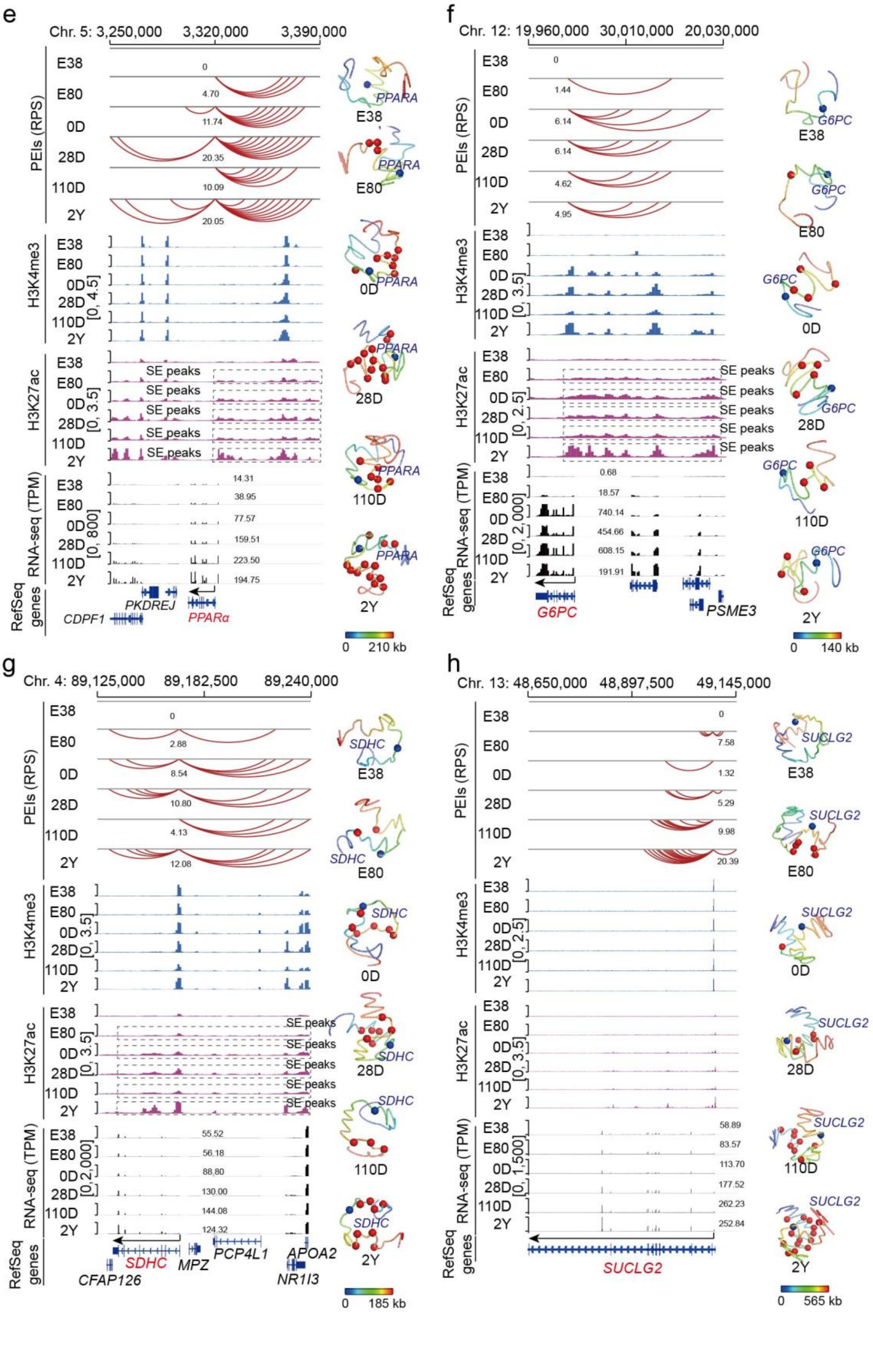

e
Chr. 5: 3,250,000 3,320,000 3,390,000
PEIs (RPS)
E38 E80 0D 28D 110D 2Y
0
4.70
11.74
20.35
10.09
20.05
H3K4me3
[0, 4.5]
H3K27ac
[0, 3.5]
SE peaks
RNA-seq (TPM)
[0, 800]
14.31
38.95
77.57
159.51
223.50
194.75
RefSeq genes
PKDREJ
CDPF1
PPARα
PPARA
E38 E80 0D 28D 110D 2Y
0 210 kb
f
Chr. 12: 19,960,000 30,010,000 20,030,000
PEIs (RPS)
E38 E80 0D 28D 110D 2Y
0
1.44
6.14
6.14
4.62
4.95
H3K4me3
[0, 3.5]
H3K27ac
[0, 2.5]
SE peaks
RNA-seq (TPM)
[0, 2,000]
0.68
18.57
740.14
454.66
608.15
191.91
RefSeq genes
G6PC
PSME3
E38 E80 0D 28D 110D 2Y
0 140 kb
g
Chr. 4: 89,125,000 89,182,500 89,240,000
PEIs (RPS)
E38 E80 0D 28D 110D 2Y
0
2.88
8.54
10.80
4.13
12.08
H3K4me3
[0, 3.5]
H3K27ac
[0, 3.5]
SE peaks
RNA-seq (TPM)
[0, 2,000]
55.52
56.18
88.80
130.00
144.08
124.32
RefSeq genes
SDHC
MPZ
PCP4L1
APOA2
CFAP126
NR1I3
E38 E80 0D 28D 110D 2Y
0 185 kb
h
Chr. 13: 48,650,000 48,897,500 49,145,000
PEIs (RPS)
E38 E80 0D 28D 110D 2Y
0
7.58
1.32
5.29
9.98
20.39
H3K4me3
[0, 2.5]
H3K27ac
[0, 3.5]
RNA-seq (TPM)
[0, 1,500]
58.89
83.57
113.70
177.52
262.23
252.84
RefSeq genes
SUCLG2
E38 E80 0D 28D 110D 2Y
0 565 kb

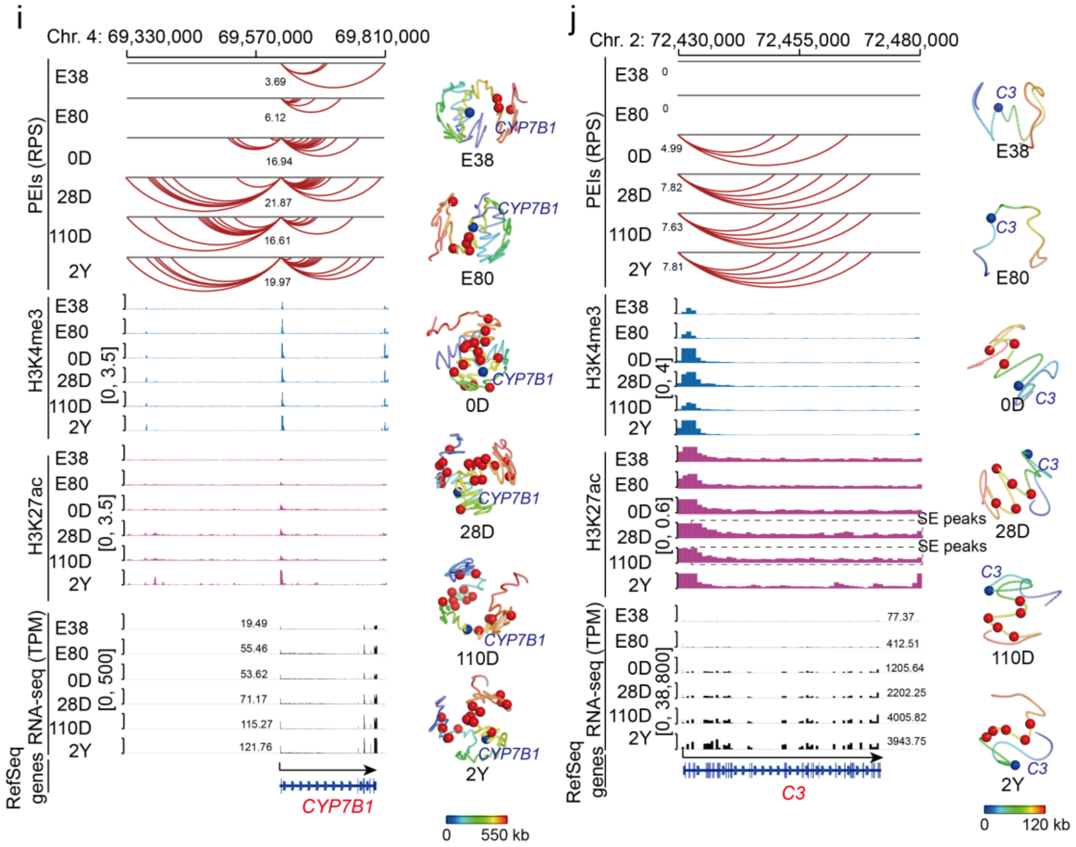

Supplementary Fig. S12 Examples of PEI rewiring related to functional transitions during liver development. a *IKZF1*, **b** *MAFB*, **c** *CPS1*, **d** *FABP1*, **e** *PPARα*, **f** *G6PC*, **g** *SDHC*, **h** *SUCLG2*, **i** *CYP7B1*, and **j** *C3*. Left: schematics of PEIs, H3K4me3 and H3K27ac signals, and transcription. Right: 3D structural models of the corresponding genomic regions. Gene promoters (blue spheres) and enhancers (red spheres) are shown.

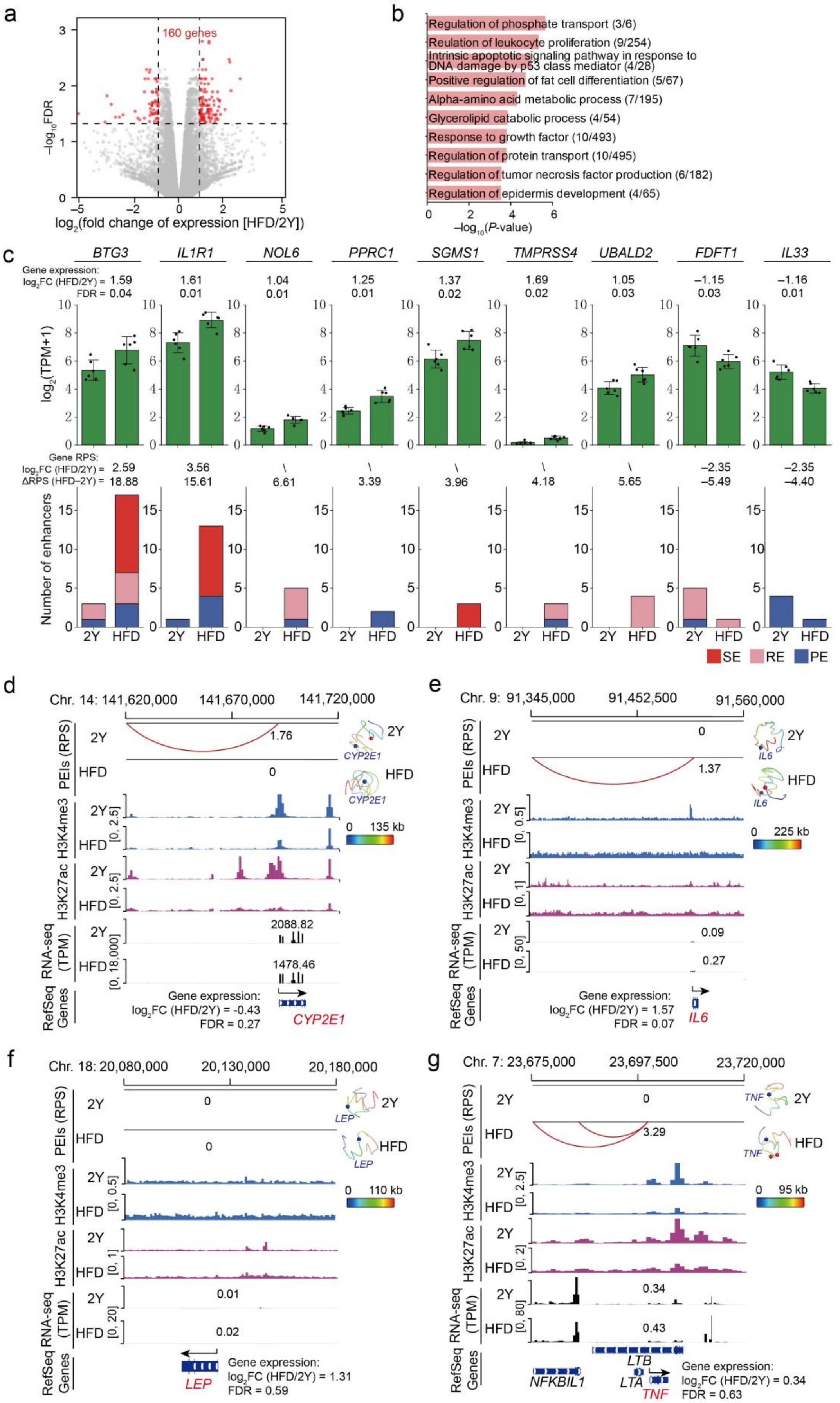

a
160 genes
$-\log_{10}$FDR
$\log_2$(fold change of expression [HFD/2Y])
b
Regulation of phosphate transport (3/6)
Reulation of leukocyte proliferation (9/254)
Intrinsic apoptotic signaling pathway in response to DNA damage by p53 class mediator (4/28)
Positive regulation of fat cell differentiation (5/67)
Alpha-amino acid metabolic process (7/195)
Glycerolipid catabolic process (4/54)
Response to growth factor (10/493)
Regulation of protein transport (10/495)
Regulation of tumor necrosis factor production (6/182)
Regulation of epidermis development (4/65)
$-\log_{10}$(P-value)
c
BTG3 IL1R1 NOL6 PPRC1 SGMS1 TMPRSS4 UBALD2 FDFT1 IL33
Gene expression: $\log_2$FC (HFD/2Y) = 1.59 1.61 1.04 1.25 1.37 1.69 1.05 –1.15 –1.16
FDR = 0.04 0.01 0.01 0.01 0.02 0.02 0.03 0.03 0.01
$\log_2$(TPM+1)
Gene RPS: $\log_2$FC (HFD/2Y) = 2.59 3.56 \ \ \ \ \ –2.35 –2.35
ΔRPS (HFD–2Y) = 18.88 15.61 6.61 3.39 3.96 4.18 5.65 –5.49 –4.40
Number of enhancers
2Y HFD
SE RE PE
d
Chr. 14: 141,620,000 141,670,000 141,720,000
PEIs (RPS) 2Y 1.76 HFD 0
CYP2E1
H3K4me3 H3K27ac RNA-seq (TPM) RefSeq Genes
2088.82
1478.46
Gene expression: $\log_2$FC (HFD/2Y) = -0.43 FDR = 0.27
e
Chr. 9: 91,345,000 91,452,500 91,560,000
PEIs (RPS) 2Y 0 HFD 1.37
IL6
0.09
0.27
Gene expression: $\log_2$FC (HFD/2Y) = 1.57 FDR = 0.07
f
Chr. 18: 20,080,000 20,130,000 20,180,000
LEP
0.01
0.02
Gene expression: $\log_2$FC (HFD/2Y) = 1.31 FDR = 0.59
g
Chr. 7: 23,675,000 23,697,500 23,720,000
TNF
3.29
0.34
0.43
LTB NFKBIL1 LTA TNF
Gene expression: $\log_2$FC (HFD/2Y) = 0.34 FDR = 0.63

Supplementary Fig. S13 Gene expression changes between HFD- and normal diet-fed pigs. a, b Identification **(a)** and functional enrichment **(b)** of genes with differential expression ($|\log_2 FC| > 1$ and FDR < 0.05) between the two groups. The enrichment analysis was conducted using Metascape[50]. We show the top ten significantly enriched terms. **c** Differences in expression, RPS, and contacting enhancers of the nine genes exhibiting concomitant changes in expression and RPS. **d–g** PEI organization, histone modifications, and expression levels of four NAFLD markers, including *CYP2E1* (**d**), *IL6* (**e**), *LEP* (**f**), and *TNF* (**g**). Left: a schematic representation of PEIs, H3K4me3 and H3K27ac signals, and transcription. Right: 3D structural models of the corresponding genomic regions. Gene promoters and enhancers are shown as blue and red spheres, respectively.

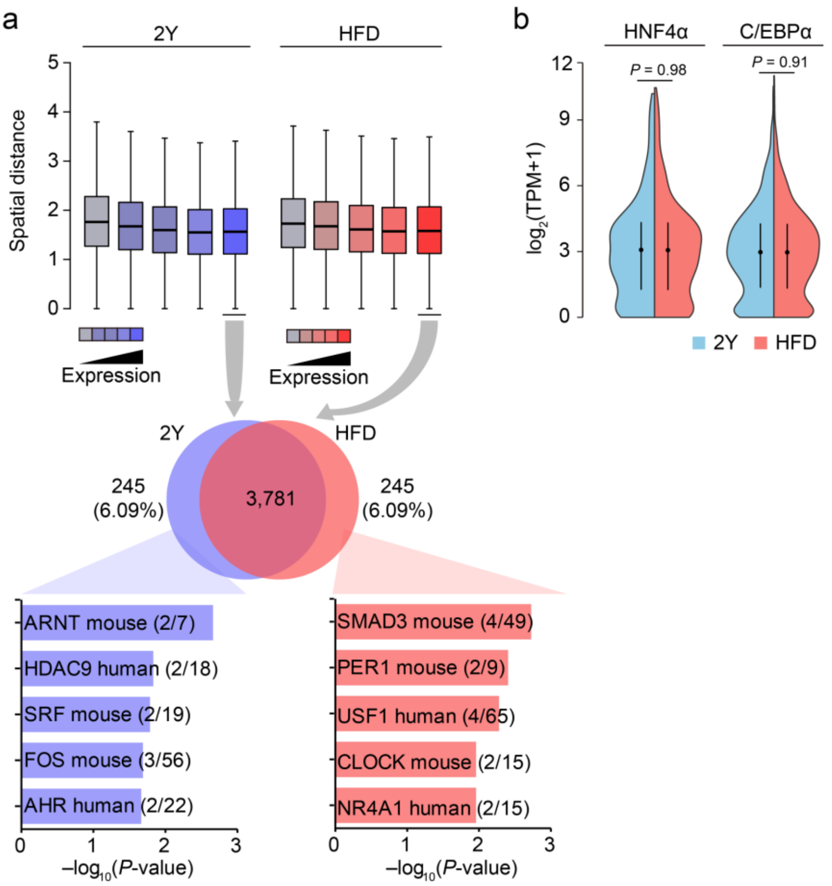

Supplementary Fig. S14 Promoter-promoter interaction profiles responding to liver metabolic stress in pigs. a Comparison of promoter-promoter interactions in liver of normally and HFD fed pigs. Top panels: 3D spatial distance among genes from different expression categories in liver of normally fed (2Y, left) and HFD fed (right) pigs. Middle panels: Overlap of the top 20% of genes with the highest expression level in liver of normally fed (2Y) and HFD fed pigs. Bottom panels: Transcription factor (TF) enrichment for the genes with specific high expression in liver of normally fed (2Y, left) and HFD fed (right) pigs. The pig genes were converted to human orthologs. TFs enriched in the specifically expressed genes were identified using the section "TRRUST Transcription Factors 2019" from the Enrichr web server (http://amp.pharm.mssm.edu/Enrichr). The top ten statistically significant TFs were depicted in bar plots. **b** Gene expression of predicted target genes of HNF4α and C/EBPα. *P*-values were calculated using a Wilcoxon rank-sum test.

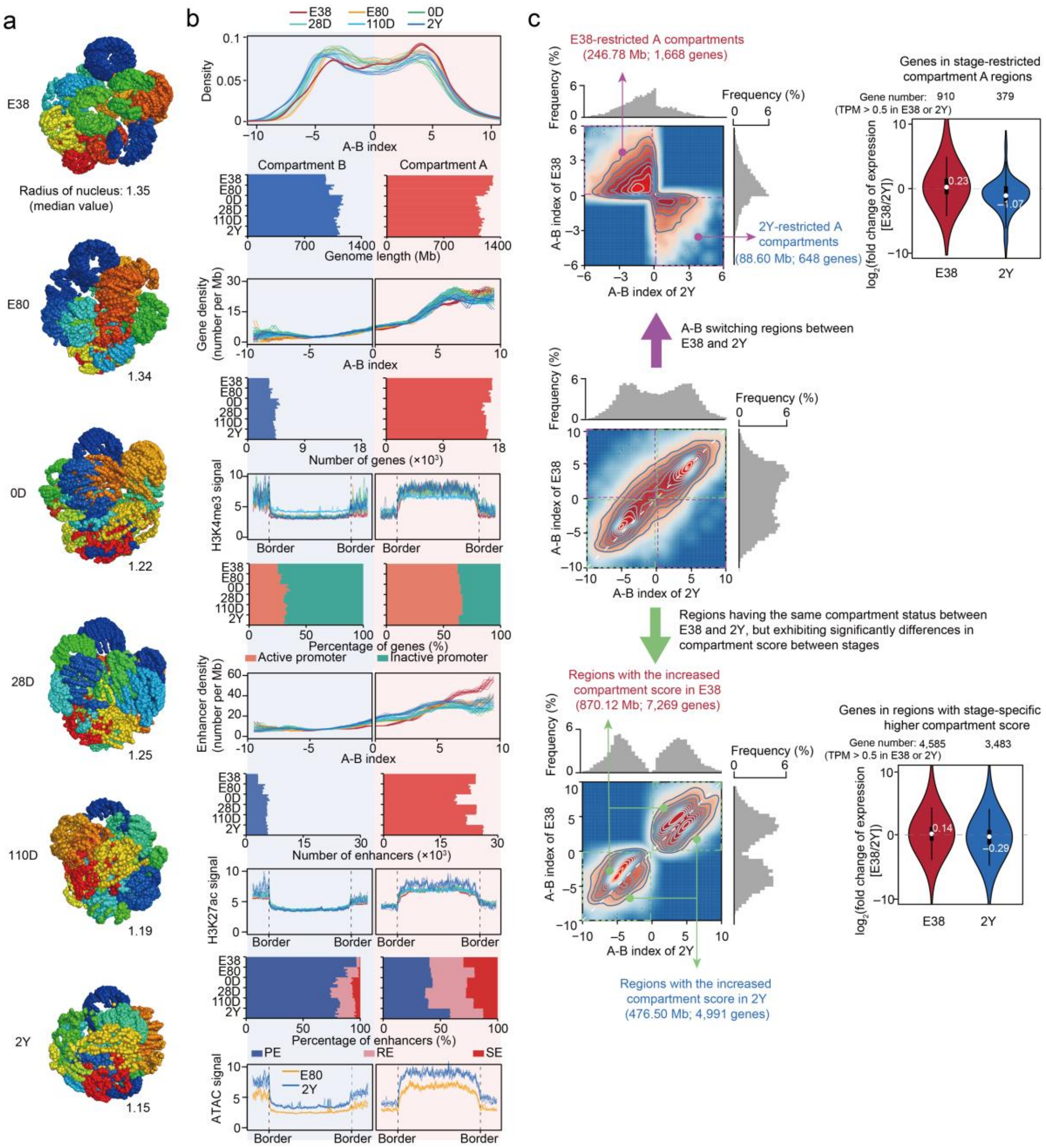

Supplementary Fig. S15 The loose chromatin architecture allows for the transcription of extensive genomic regions during early liver development. a 3D genome structures of porcine livers during development, which were inferred using the normalized intra- (20-kb resolution) and inter-chromosomal (1-Mb resolution) contact maps with the miniMDS[96] and visualized with PyMOL (v 2.5.2). The nuclear radius is determined by the average distance to the nuclear center of mass. **b** Comparison of genomic features between regions that have distinct compartment status (A or B) during liver development, including compartment length, number and density of genes, enhancer density, as well as H3K4me3 ChIP-seq, H3K27ac ChIP-seq and ATAC-seq signals. **c** Expansion of the transcriptionally active compartment A regions facilitate widespread active transcription during early liver development. By comparing the representative stages between prenatal E38 and adult 2Y stages, we observed that 246.78 Mb (10.89% of the genome) and 88.60 Mb (3.91% of the genome) specifically exhibited compartment A status in E38 and 2Y, respectively. The genes located in

these stage-restricted compartment A regions (1,668 for E38, and 648 for 2Y) tended to show increased gene expression. We then compared compartment scores (i.e., the A-B index values) for the regions with the same compartment status between the two stages. This allowed us to identify regions spanning 870.12 Mb (38.40% of the genome) and 476.50 Mb (21.03% of the genome) that exhibited statistically significant elevated compartment scores ($P < 0.05$, unpaired Student's *t*-test, and $|\triangle$A-B index$| > 0.5$) in E38 and 2Y, respectively. The genes located in these stage-specific higher compartment score regions (7,269 for E38, and 4,991 for 2Y) also tended to show increased gene expression. The genes with evidence of transcription (TPM > 0.5) in E38 or 2Y stages were used for expression comparison.

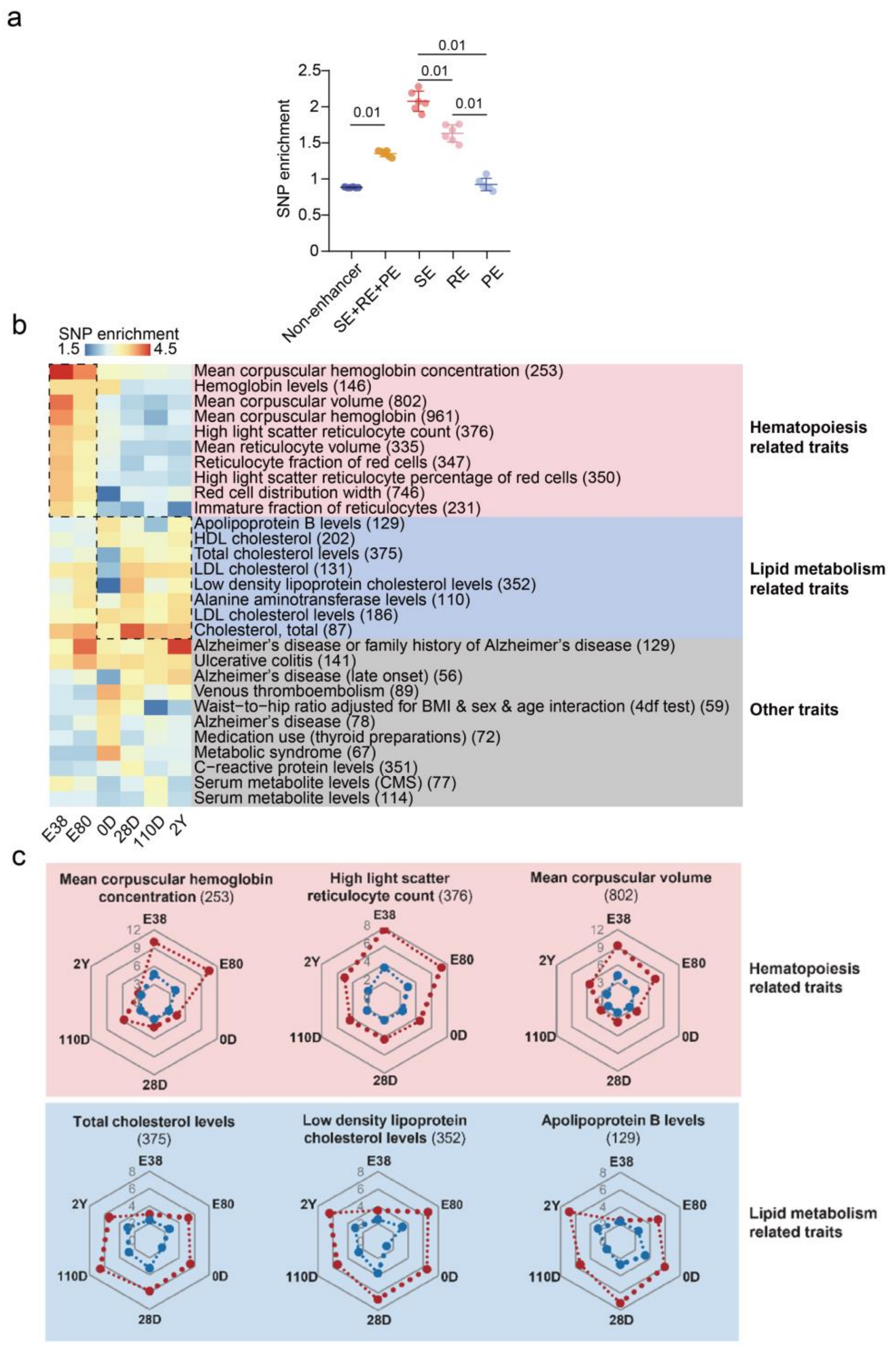

Supplementary Fig. S16 Human trait-associated noncoding SNPs were enriched in enhancers in the porcine liver. a Comparison of trait-associated, noncoding SNP enrichments in enhancers and other genomic regions in the porcine liver across six developmental stages. The identification of SEs, REs, and PEs was based on H3K27ac binding peaks. SNP enrichment score calculation can be found in **Materials and methods**. **b** Heatmap of noncoding SNP enrichment scores in enhancer regions in the porcine liver across six developmental stages. The merged traits or diseases with the top ten highest SNP enrichment scores at each stage (P < 0.05, χ^2 test) are displayed. Traits or diseases can be generally classified into three categories:

hematopoiesis-related traits, lipid metabolism-related traits, and others. The numbers in parentheses indicate the counts of trait-associated, noncoding SNPs. **c** Radar plots showing differences in SNP enrichment linked to traits of core liver functions, including hematopoiesis (upper) and lipid metabolism (lower), in enhancers during six developmental stages of the porcine liver. The colored dots on the axis indicate SNP enrichment scores in SEs (red points) or the union set of all enhancers (blue points) at each stage. The numbers in parentheses indicate the counts of trait-associated, noncoding SNPs.

Supplementary Tables

Supplementary Table S1 Functional description of the genes mentioned in this study.

Gene symbol	Gene name	Related figure in manuscript	Gene function	Reference
HBB*, *HBE1	Hemoglobin subunit beta, Hemoglobin subunit epsilon 1	Fig. 3b; Supplementary Fig. S6b	These genes encode 146-amino-acid globin chains, which compose hemoglobin in combination with α-like globin to participate in oxygen transport.	Patrinos, G. P., Kollia, P. & Papadakis, M. N. Molecular diagnosis of inherited disorders: lessons from hemoglobinopathies. *Hum Mutat* **26**, 399-412 (2005).<br>Keys, J. R. *et al.* A mechanism for Ikaros regulation of human globin gene switching. *Br J Haematol* **141**, 398-406 (2008).
SGCE	Sarcoglycan epsilon	Fig. 3c; Supplementary Fig. S6b	A maternally imprinted gene that is broadly expressed in embryos. Mutants in this gene result in myoclonus-dystonia syndrome. It is highly expressed in several tumours, including breast cancer stem cells and HCC.	Piras, G. et al. *Zac1* (*Lot1*), a potential tumor suppressor gene, and the gene for epsilon-sarcoglycan are maternally imprinted genes: identification by a subtractive screen of novel uniparental fibroblast lines. *Mol Cell Biol* **20**, 3308-3315 (2000).<br>Grabowski, M. et al. The epsilon-sarcoglycan gene (*SGCE*), mutated in myoclonus-dystonia syndrome, is maternally imprinted. *Eur J Hum Genet* **11**, 138-144 (2003).<br>Zhao, L. et al. *SGCE* promotes breast cancer stem cells by stabilizing EGFR. *Adv Sci* **7**, 1903700 (2020).<br>Dong, H. et al. Gene expression profile analysis of human hepatocellular carcinoma using SAGE and

				LongSAGE. *BMC Med Genomics* **2**, 5 (2009).
PEG10	Paternally expressed 10	Fig. 3c; Supplementary Fig. S6b	A maternally imprinted gene. Its deletion causes early embryonic lethality, and its overexpression promotes breast cancer, HCC and liver regeneration under regulation of c-MYC.	Ono, R. et al. Deletion of *PEG10*, an imprinted gene acquired from a retrotransposon, causes early embryonic lethality. *Nat Genet* **38**, 101-106 (2006).
				Li, X. et al. *PEG10* promotes human breast cancer cell proliferation, migration and invasion. *Int J Oncol* **48**, 1933-1942 (2016).
				Tsou, A. P. et al. Overexpression of a novel imprinted gene, *PEG10*, in human hepatocellular carcinoma and in regenerating mouse livers. *J Biomed Sci* **10**, 625-635 (2003).
				Li, C. M. et al. *PEG10* is a c-MYC target gene in cancer cells. *Cancer Res* **66**, 665-672, (2006).
PDK4	Pyruvate dehydrogenase kinase 4	Fig. 3c; Supplementary Fig. S6b	A protein kinase, which phosphorylates and inactivates pyruvate dehydrogenase complex that catalyzes a rate-limiting step of glucose oxidation. Its upregulated expression is associated with fatty acid oxidation in liver and heart.	Kim, Y. I., Lee, F. N., Choi, W. S., Lee, S. & Youn, J. H. Insulin regulation of skeletal muscle *PDK4* mRNA expression is impaired in acute insulin-resistant states. *Diabetes* **55**, 2311-2317 (2006).
				Pettersen, I. K. N. et al. Upregulated *PDK4* expression is a sensitive marker of increased fatty acid oxidation. *Mitochondrion* **49**, 97-110 (2019).
PON1, PON2, PON3	Paraoxonase-1, -2, -3	Fig. 3c, 5, 6b; Supplementary Fig. S6b	They are members of paraoxonase gene family that are responsible for protecting cells from oxidative stress. All of them can prevent the oxidation of LDL and thus have the anti-atherogenic capacity. PON1	Rajkovic, M. G., Rumora, L. & Barisic, K. The paraoxonase 1, 2 and 3 in humans. *Biochem Med* **21**, 122-130 (2011).
				Reddy, S. T., Devarajan, A., Bourquard, N., Shih, D. & Fogelman, A. M. Is it just paraoxonase 1 or are other

			has paraoxonase and arylesterase activities and can hydrolyse different kinds of substrates, while PON2 and PON3 can process very limited kinds of substrates.	members of the paraoxonase gene family implicated in atherosclerosis? *Curr Opin Lipidol* **19**, 405-408 (2008).
SOX6	SRY-box transcription factor 6	Fig. 3e; Supplementary Fig. S6c	It is highly expressed in proerythroblasts and erythroblasts in the fetal liver and encodes a transcription factor that stimulates erythroid cell survival, proliferation, and terminal maturation while silences epsilon globin expression in definitive erythropoiesis.	Dumitriu, B. et al. *SOX6* cell-autonomously stimulates erythroid cell survival, proliferation, and terminal maturation and is thereby an important enhancer of definitive erythropoiesis during mouse development. *Blood* **108**, 1198-1207 (2006).
				Yi, Z. et al. *SOX6* directly silences epsilon globin expression in definitive erythropoiesis. *PLoS Genet* **2**, e14 (2006)
				Cohen-Barak, O. et al. Stem cell transplantation demonstrates that *SOX6* represses epsilon y globin expression in definitive erythropoiesis of adult mice. *Exp Hematol* **35**, 358-367 (2007).
RHAG	Rh associated glycoprotein	Supplementary Fig. S6b, S7a	It encodes a glycoprotein that is specifically expressed in erythroid surface. Absence of this glycoprotein in Rh_{null} individuals leads to chronic haemolytic anaemia. RhAG may participate in NH_3 and CO_2 gas transport in red cells.	Marini, A. M. et al. The human Rhesus-associated RHAG protein and a kidney homologue promote ammonium transport in yeast. *Nat Genet* **26**, 341-344 (2000).
				Ripoche, P. et al. Role of *RHAG* and *AQP1* in NH_3 and CO_2 gas transport in red cell ghosts: a stopped-flow analysis. *Transfus Clin Biol* **13**, 117-122 (2006).

SPTA1	Spectrin alpha	Supplementary Fig. S6b, S7b	*SPTA1* encodes α-spectrin, the predominant constituent of cell membrane skeleton in red blood. Mutations in α-spectrin are associated with hereditary spherocytosis.	Wichterle, H., Hanspal, M., Palek, J. & Jarolim, P. Combination of two mutant alpha spectrin alleles underlies a severe spherocytic hemolytic anemia. *J Clin Invest* **98**, 2300-2307 (1996).<br>Chonat, S. et al. The spectrum of *SPTA1*-associated hereditary spherocytosis. *Front Physiol* **10**, 815 (2019).
CDK1	Cyclin dependent kinase 1	Supplementary Fig. S6b, S7c	This gene encodes an archetypical kinase, which serves as a central regulator binding to all cyclins to promote cell cycle. Overexpression of CDK1 is sufficient to initiate HCC.	Santamaría, D. et al. *CDK1* is sufficient to drive the mammalian cell cycle. *Nature* **448**, 811-815 (2007).<br>Deane, N. G. et al. Hepatocellular carcinoma results from chronic *Cyclin D1* overexpression in transgenic mice. *Cancer Res* **61**, 5389-5395 (2001).<br>Joo, M., Kang, Y. K., Kim, M. R., Lee, H. K. & Jang, J. J. *Cyclin D1* overexpression in hepatocellular carcinoma. *Liver* **21**, 89-95 (2001).
CENPW	Centromere protein W	Supplementary Fig. S6b, S7d	CENPW, identified as a centromeric component, plays a central role in assembly of kinetochore and mitotic chromosome segregation. It is also known as a cancer upregulated gene 2 (CUG2) which is overexpressed in various human cancers, such as HCC.	Hori, T. et al. CCAN makes multiple contacts with centromeric DNA to provide distinct pathways to the outer kinetochore. *Cell* **135**, 1039-1052 (2008).<br>Zhou, Z., Zhou, Z., Huang, Z., He, S. & Chen, S. Histone-fold centromere protein W (*CENP-W*) is associated with the biological behaviour of hepatocellular carcinoma cells. *Bioengineered* **11**, 729-742 (2020).

HMGB2	High mobility group box 2	Supplementary Fig. S6b, S7e	*HMGB2* is highly expressed during embryogenesis and associated with chondrogenesis, spermatogenesis. It is overexpressed in a variety of human cancers, including gastric cancer and HCC.	Taniguchi, N. et al. Chromatin protein HMGB2 regulates articular cartilage surface maintenance *via* beta-catenin pathway. *Proc Natl Acad Sci U S A* **106**, 16817-16822 (2009).
				Ronfani, L. et al. Reduced fertility and spermatogenesis defects in mice lacking chromosomal protein HMGB2. *Development* **128**, 1265-1273 (2001).
				Cui, G., Cai, F., Ding, Z. & Gao, L. *HMGB2* promotes the malignancy of human gastric cancer and indicates poor survival outcome. *Hum Pathol* **84**, 133-141 (2019).
				Kwon, J. H. et al. Overexpression of high-mobility group box 2 is associated with tumor aggressiveness and prognosis of hepatocellular carcinoma. *Clin Cancer Res* **16**, 5511-5521 (2010).
GYS2	Glycogen synthase 2	Supplementary Fig. S6b, S7f	It encodes a rate-limiting enzyme for glycogen synthesis. It is predominantly expressed in the liver, whose mutation results in Glycogen Storage Disease Type 0 (GSD0).	Kadotani, A. et al. Metabolic impact of overexpression of liver glycogen synthase with serine-to-alanine substitutions in rat primary hepatocytes. *Arch Biochem Biophys* **466**, 283-289 (2007).
				Nessa, A. et al. Mutational analysis of the *GYS2* gene in patients diagnosed with ketotic hypoglycaemia. *J Pediatr Endocrinol Metab* **25**, 963-967 (2012).
PAH	Phenylalanine hydroxylase	Supplementary Fig. S6b, S7g	It encodes a rate-limiting enzyme of phenylalanine catabolism which catalyses the conversion of L-	Flydal, M. I. & Martinez, A. Phenylalanine hydroxylase: function, structure, and regulation. *IUBMB life* **65**, 341-349 (2013).

			phenylalanine to L-tyrosine. PAH deficiency results in intolerance to the dietary intake of the essential amino acid phenylalanine and leads to phenylketonuria.	Mitchell, J. J., Trakadis, Y. J. & Scriver, C. R. Phenylalanine hydroxylase deficiency. *Genet Med* **13**, 697-707 (2011).
GHR	Growth hormone receptor	Supplementary Fig. S6b, S7h	GHR is a key regulator of postnatal growth and is closely related to metabolism. GH binds to GHR, followed by the activation of the JAK-STAT pathway and subsequent increase in expression of *IGF1*.	Brooks, A. J., Wooh, J. W., Tunny, K. A. & Waters, M. J. Growth hormone receptor; mechanism of action. *Int J Biochem Cell Biol* **40**, 1984-1989 (2008).
				Dos Santos, C. et al. A common polymorphism of the growth hormone receptor is associated with increased responsiveness to growth hormone. *Nat Genet* **36**, 720-724 (2004).
IGF1	Insulin like growth factor 1	Supplementary Fig. S6b, S7i	IGF1 is primarily produced by the liver hepatocytes and involved in regulation of growth and metabolism.	Kineman, R. D., Del Rio-Moreno, M. & Sarmento-Cabral, A. 40 years of *IGF1*: Understanding the tissue-specific roles of *IGF1/IGF1R* in regulating metabolism using the Cre/loxP system. *J Mol Endocrinol* **61**, t187-t198 (2018).
				Stratikopoulos, E., Szabolcs, M., Dragatsis, I., Klinakis, A. & Efstratiadis, A. The hormonal action of *IGF1* in postnatal mouse growth. *Proc Natl Acad Sci U S A* **105**, 19378-19383 (2008).
CCL5	C-C motif chemokine ligand 5	Supplementary Fig. S6b, S7j	*CCL5* encodes a chemokine ligand that is correlated with the promotion of cell survival, proliferation and invasion by binding to CCR5.	Weng, C. J. et al. Effect of CC chemokine ligand 5 and CC chemokine receptor 5 genes polymorphisms on the risk and clinicopathological development of oral cancer. *Oral Oncol* **46**, 767-772 (2010).

			Mutations in *CCL5* are significantly associated with HCC.	Tsai, H. T., Yang, S. F., Chen, D. R. & Chan, S. E. *CCL5-28*, *CCL5-403*, and *CCR5* genetic polymorphisms and their synergic effect with alcohol and tobacco consumptions increase susceptibility to hepatocellular carcinoma. *Med Oncol* **29**, 2771-2779 (2012).
CCL14	C-C motif chemokine ligand 14	Supplementary Fig. S6b, S7j	CCL14 is a chemokine that promotes the activation of immune cells by binding to CCR1, as well as promotes chemotaxis of monocytes, eosinophils, and T lymphoblasts. It inhibits the growth of liver tumour by inhibiting the activation of Wnt/β-catenin pathway and can serve as a prognostic biomarker of HCC.	Nagarsheth, N., Wicha, M. S. & Zou, W. Chemokines in the cancer microenvironment and their relevance in cancer immunotherapy. *Nat Rev Immunol* **17**, 559-572 (2017).
				Zhu, M. et al. *CCL14* serves as a novel prognostic factor and tumor suppressor of HCC by modulating cell cycle and promoting apoptosis. *Cell Death Dis* **10**, 796 (2019).
				Gu, Y. et al. *CCL14* is a prognostic biomarker and correlates with immune infiltrates in hepatocellular carcinoma. *Aging* **12**, 784-807 (2020).
IL1R1	Interleukin 1 receptor type 1	Supplementary Fig. S6b, S7k	IL-1R1 is a membrane receptor belonging to the IL-1R family. IL-1 ligands bind to IL-1R1 and the complex is responsible for IL-1-mediated inflammatory activation and liver tumorigenesis.	Boraschi, D. & Tagliabue, A. The interleukin-1 receptor family. *Semin Immunol* **25**, 394-407 (2013).
				Garlanda, C., Dinarello, C. A. & Mantovani, A. The interleukin-1 family: back to the future. *Immunity* **39**, 1003-1018 (2013).
				Sakurai, T. et al. Hepatocyte necrosis induced by oxidative stress and IL-1 alpha release mediate carcinogen-induced compensatory proliferation and liver tumorigenesis. *Cancer cell* **14**, 156-165 (2008).

LIN28B	Lin-28 homolog B	Supplementary Fig. S6c, S7l	This gene is specifically expressed in hepatoblasts and maintains the stem cell properties of hepatoblasts, such as proliferation and high self-renewal potential of fetal hematopoietic stem cells.	Takashima, Y. et al. Suppression of lethal-7b and miR-125a/b Maturation by *LIN28B* Enables Maintenance of Stem Cell Properties in Hepatoblasts. *Hepatology* **64**, 245-260 (2016).
				Copley, M. R. et al. The *LIN28b*-let-7-*HMGA2* axis determines the higher self-renewal potential of fetal haematopoietic stem cells. *Nat Cell Biol* **15**, 916-925 (2013).
ABRACL	ABRA C-terminal like	Fig. 4d	It is previously named as *HSCP280*. It is associated with tumorigenesis and proliferation.	Wang, D., Liu, H., Ren, C. & Wang, L. High expression of *ABRACL* is associated with tumorigenesis and affects clinical outcome in gastric cancer. *Genet Test Mol Biomarkers* **23**, 91-97 (2019).
				Ura, B. et al. A proteomic approach for the identification of biomarkers in endometrial cancer uterine aspirate. *Oncotarget* **8**, 109536-109545 (2017).
ADH4	Alcohol dehydrogenase 4	Fig. 5; Supplementary Fig. S10c	ADH4 is a member of ADH family that metabolizes a wide variety of substrates including ethanol and retinol. The gene expression level of *ADH4* is negatively correlated with HCC, while the variations of *ADH4* are closely correlated to high risk of alcohol dependence and drug dependence in a recessive manner.	Wei, R. R. et al. Identification of *ADH4* as a novel and potential prognostic marker in hepatocellular carcinoma. *Med Oncol* **29**, 2737-2743 (2012).
				Deltour, L., Foglio, M. H. & Duester, G. Impaired retinol utilization in *ADH4* alcohol dehydrogenase mutant mice. *Dev Genet* **25**, 1-10 (1999).
				Luo, X. et al. *ADH4* gene variation is associated with alcohol dependence and drug dependence in European Americans: results from HWD tests and case-control association studies.

				Neuropsychopharmacology **31**, 1085-1095 (2006).
FGL2	Fibrinogen like 2	Supplementary Fig. S10d	FGL2 is an inducible prothrombinase that plays an important role in the pathogenesis of fibrin deposition during viral hepatitis and significantly increased expression in hepatitis C virus patients.	Marsden, P. A. et al. The *FGL2/fibroleukin* prothrombinase contributes to immunologically mediated thrombosis in experimental and human viral hepatitis. *J Clin Invest* **112**, 58-66 (2003).
				Ghanekar, A. et al. Endothelial induction of *FGL2* contributes to thrombosis during acute vascular xenograft rejection. *J Immunol* **172**, 5693-5701 (2004).
				Foerster, K. et al. The novel immunoregulatory molecule *FGL2*: a potential biomarker for severity of chronic hepatitis C virus infection. *J Hepatol* **53**, 608-615 (2010).
POSTN	Periostin	Supplementary Fig. S10e	POSTN promotes the expression of TGFb1 and HIF1a to facilitate angiogenesis, cellular survival, migration and resistance to hypoxia-induced cell death. The overexpression of POSTN is observed in many cancers, including glioma, ovarian carcinoma, and colorectal cancer.	Oh, H. J. et al. Overexpression of *POSTN* in tumor stroma is a poor prognostic indicator of colorectal cancer. *J Pathol Transl Med* **51**, 306-313 (2017).
				Park, S. Y., Piao, Y., Jeong, K. J., Dong, J. & de Groot, J. F. Periostin (*POSTN*) regulates tumor resistance to antiangiogenic therapy in glioma models. *Mol Cancer Ther* **15**, 2187-2197 (2016).
				Sung, P. L. et al. *Periostin* in tumor microenvironment is associated with poor prognosis and platinum resistance in epithelial ovarian carcinoma. *Oncotarget* **7**, 4036-4047 (2016).

IKZF1	Ikaros family zinc finger 1	Fig. 5; Supplementary Fig. S12a	This gene is also known as *IKAROS*, which encodes a lineage-regulating factor in the hematopoietic system.	Georgopoulos, K. et al. The Ikaros gene is required for the development of all lymphoid lineages. *Cell* **79**, 143-156 (1994).
				Georgopoulos, K. Haematopoietic cell-fate decisions, chromatin regulation and Ikaros. *Nat Rev Immunol* **2**, 162-174 (2002).
				Yoshida, T., Ng, S. Y., Zuniga-Pflucker, J. C. & Georgopoulos, K. Early hematopoietic lineage restrictions directed by Ikaros. *Nat Immunol* **7**, 382-391 (2006).
MAFB	MAF bZIP transcription factor B	Fig. 5; Supplementary Fig. S12b	MAFB is highly expressed in myelomonocyte but not in erythroid cells, which represses erythroid differentiation by interacting with Ets-1 while stimulates macrophage differentiation.	Sieweke, M. H., Tekotte, H., Frampton, J. & Graf, T. *MAFB* is an interaction partner and repressor of *ETS-1* that inhibits erythroid differentiation. *Cell* **85**, 49-60 (1996).
				Kelly, L. M., Englmeier, U., Lafon, I., Sieweke, M. H. & Graf, T. *MAFB* is an inducer of monocytic differentiation. *Embo J* **19**, 1987-1997 (2000).
CPS1	Carbamoyl-phosphate synthase 1	Fig. 5; Supplementary Fig. S12c	CPS1 is specifically expressed in liver and is the first rate-limiting enzyme in the urea cycle. Expression of *CPS1* is decreased in HCC and NAFLD due to the hyper-methylation of promoter, resulting in urea cycle dysregulation.	Liu, H., Dong, H., Robertson, K. & Liu, C. DNA methylation suppresses expression of the urea cycle enzyme carbamoyl phosphate synthetase 1 (*CPS1*) in human hepatocellular carcinoma. *Am J Pathol* **178**, 652-661 (2011).
				De Chiara, F. et al. Urea cycle dysregulation in non-alcoholic fatty liver disease. *J Hepatol* **69**, 905-915 (2018).

FABP1	Fatty acid binding protein 1	Fig. 5; Supplementary Fig. S12d	FABP1 is highly expressed in the liver and facilitates uptake, transport, mitochondrial oxidation, and esterification of fatty acids. FABP1 level is positively correlated with NAFLD and knockdown of *FABP1* promotes NAFLD.	Atshaves, B. P. et al. Liver fatty acid-binding protein gene ablation inhibits branched-chain fatty acid metabolism in cultured primary hepatocytes. *J Biol Chem* **279**, 30954-30965 (2004)<br>Atshaves, B. P. et al. Liver fatty acid-binding protein and obesity. *J Nutr Biochem* **21**, 1015-1032 (2010).<br>Lu, Y. C. et al. Circulating fatty acid-binding protein 1 (*FABP1*) and nonalcoholic fatty liver disease in patients with type 2 diabetes mellitus. *Int J Med Sci* **17**, 182-190 (2020).<br>Mukai, T., Egawa, M., Takeuchi, T., Yamashita, H. & Kusudo, T. Silencing of *FABP1* ameliorates hepatic steatosis, inflammation, and oxidative stress in mice with nonalcoholic fatty liver disease. *FEBS Open Bio* **7**, 1009-1016 (2017).
PPARα	Peroxisome proliferator activated receptor alpha	Fig. 5; Supplementary Fig. S12e	PPAR*α* is a member of PPAR family, which is abundantly expressed in the liver and is essential for modulation of lipid transport and metabolism through transcriptionally activated genes that are involved in mitochondrial and peroxisomal fatty acid β-oxidation. The absence of PPAR*α* enhances hepatic steatosis.	Reddy, J. K. Peroxisome proliferators and peroxisome proliferator-activated receptor alpha: biotic and xenobiotic sensing. *Am J Pathol* **164**, 2305-2321 (2004).<br>Abdelmegeed, M. A. et al. *PPARα* expression protects male mice from high fat-induced nonalcoholic fatty liver. *J Nutr* **141**, 603-610 (2011).<br>Brocker, C. N. et al. Extrahepatic *PPARα* modulates fatty acid oxidation and attenuates fasting-induced hepatosteatosis in mice. *J Lipid Res* **59**, 2140-2152 (2018).

G6PC	Glucose-6-phosphatase catalytic subunit	Fig. 5; Supplementary Fig. S12f	G6PC is a key enzyme in the maintenance of glucose homeostasis, which catalyses the hydrolysis of glucose-6-phosphate to glucose and phosphate in the terminal step of gluconeogenesis and glycogenolysis. The mutations in *G6PC* are associated with Glycogen storage disease type Ia.	Chou, J. Y. & Mansfield, B. C. Mutations in the glucose-6-phosphatase-alpha (*G6PC*) gene that cause type Ia glycogen storage disease. *Hum Mutat* **29**, 921-930 (2008).<br>Zheng, B. X., Lin, Q., Li, M. & Jin, Y. Three novel mutations of the *G6PC* gene identified in Chinese patients with glycogen storage disease type Ia. *Eur J Pediatr* **174**, 59-63 (2015).
SDHC	Succinate dehydrogenase complex subunit C	Fig. 5; Supplementary Fig. S12g	SDHC is a member of succinate dehydrogenase (SDH) complex that catalyses the oxidation of succinate to fumarate in the Krebs cycle and the respiratory chain. Mutations in SDHC cause increased $O2^-$ production that can contribute to nuclear DNA damage, mutagenesis, and ultimately, tumorigenesis.	Rustin, P., Munnich, A. & Rötig, A. Succinate dehydrogenase and human diseases: new insights into a well-known enzyme. *Eur J Hum Genet* **10**, 289-291 (2002).<br>Slane, B. G. et al. Mutation of succinate dehydrogenase subunit C results in increased O_2.-, oxidative stress, and genomic instability. *Cancer Res* **66**, 7615-7620 (2006).<br>Ishii, T. et al. A mutation in the *SDHC* gene of complex II increases oxidative stress, resulting in apoptosis and tumorigenesis. *Cancer Res* **65**, 203-209 (2005).
SUCLG2	Succinate-CoA ligase GDP-forming subunit beta	Fig. 5; Supplementary Fig. S12h	SUCLG2 is a subunit of succinate-CoA ligase (SUCL) at the crossroad of several biochemical pathways encompassing the citric acid cycle	Kacso, G. et al. Two transgenic mouse models for β-subunit components of succinate-CoA ligase yielding pleiotropic metabolic alterations. *Biochem J* **473**, 3463-3485 (2016).

			and metabolism of ketone bodies. Knockdown of *SUCLG2* resulted in decrease in mtDNA amount.	Miller, C., Wang, L., Ostergaard, E., Dan, P. & Saada, A. The interplay between *SUCLA2*, *SUCLG2*, and mitochondrial DNA depletion. *Biochim Biophys Acta* **1812**, 625-629 (2011).
CYP7B1	Cytochrome P450 family 7 subfamily B member 1	Fig. 5; Supplementary Fig. S12i	CYP7B1 is a member of cytochrome P450 superfamily, which is highly expressed in the liver and is crucial for the inactivation of oxysterols and their subsequent conversion into bile salts.	Stiles, A. R., McDonald, J. G., Bauman, D. R. & Russell, D. W. *CYP7B1*: one cytochrome P450, two human genetic diseases, and multiple physiological functions. *J Biol Chem* **284**, 28485-28489 (2009).
C3	Complement C3	Fig. 5; Supplementary Fig. S12j	C3 is a member of complement system and plays a central role in the complement cascade, which is essential to the innate immune process.	Thorgersen, E. B. et al. The role of complement in liver injury, regeneration, and transplantation. *Hepatology* **70**, 725-736 (2019).
				Qin, X. & Gao, B. The complement system in liver diseases. *Cell Mol Immunol* **3**, 333-340 (2006).
TAL1	TAL bHLH transcription factor 1	Fig. 5, 6a	TAL1, also known as SCL, is a master regulator of hematopoiesis and is a crucial oncogene in T-cell leukemogenesis.	Vagapova, E. R., Spirin, P. V., Lebedev, T. D. & Prassolov, V. S. The role of *TAL1* in hematopoiesis and leukemogenesis. *Acta Naturae* **10**, 15-23 (2018).
				Sanda, T. & Leong, W. Z. *TAL1* as a master oncogenic transcription factor in T-cell acute lymphoblastic leukemia. *Exp Hematol* **53**, 7-15 (2017).
ADIPOQ	Adiponectin	Fig. 7f	ADIPOQ is highly expressed in adipose tissue, and correlates with hepatic fat content and insulin resistance.	Buechler, C., Wanninger, J. & Neumeier, M. Adiponectin, a key adipokine in obesity related liver diseases. *World J Gastroenterol* **17**, 2801-2811 (2011).

				Westerbacka, J. et al. Women and men have similar amounts of liver and intra-abdominal fat, despite more subcutaneous fat in women: implications for sex differences in markers of cardiovascular risk. *Diabetologia* **47**, 1360-1369 (2004).
CYP2E1	Cytochrome P450 Family 2 Subfamily E Member 1	Supplementary Fig. S13d	CYP2E1, a pro-oxidase, is crucial in lipid peroxides and highly expressed in NAFLD patients.	Bell, L. N. et al. Hepatic lipid peroxidation and cytochrome P450 2E1 in pediatric nonalcoholic fatty liver disease and its subtypes. *J Clin Gastroenterol* **45**, 800-807 (2011).
IL6	Interleukin 6	Supplementary Fig. S13e	IL-6 is a multifunctional cytokine which regulates immune responses. Hepatic IL6 expression correlates with different aspects of NAFLD.	Tilg, H. The role of cytokines in non-alcoholic fatty liver disease. *Dig Dis* **28**, 179-185 (2010).
LEP	Leptin	Supplementary Fig. S13f	LEP, a non-glycosylated protein, is secreted by adipocytes and hepatic stellate cells. Increased LEP serum level is correlated with steatohepatitis.	Bethanis, S. K. & Theocharis, S. E. Leptin in the field of hepatic fibrosis: a pivotal or an incidental player? *Dig Dis* **51**, 1685-1696 (2006).
				Uygun, A. et al. Serum *leptin* levels in patients with nonalcoholic steatohepatitis. *Am J Gastroenterol* **95**, 3584-3589 (2000).
				Chitturi, S. et al. Serum *leptin* in NASH correlates with hepatic steatosis but not fibrosis: a manifestation of lipotoxicity? *Hepatology* **36**, 403-409 (2002).
TNF	Tumor Necrosis Factor	Supplementary Fig. S13g	TNFa, a proinflammatory cytokine, plays roles in the progression of NAFLD and significantly increases	Jarrar, M. H. et al. Adipokines and cytokines in non-alcoholic fatty liver disease. *Aliment Pharmacol Ther* **27**, 412-421 (2008).

			in different rodent obesity models.	Bahcecioglu, I. H. et al. Levels of serum hyaluronic acid, *TNF-alpha* and *IL-8* in patients with nonalcoholic steatohepatitis. *Hepatogastroenterology* **52**, 1549-1553 (2005).
				Xu, H., Uysal, K. T., Becherer, J. D., Arner, P. & Hotamisligil, G. S. Altered tumor necrosis factor-alpha (*TNF-alpha*) processing in adipocytes and increased expression of transmembrane *TNF-alpha* in obesity. *Diabetes* **51**, 1876-1883 (2002).

Supplementary Table S2 Information of primers and genomic locations for target gene promoters and enhancers validated using the Dual-Luciferase reporter assay.

Gene symbol	Promoter/Enhancer symbol	Promoter/Enhancer region	PCR position	Amplicon length (bp)	Forward primer (5'-3')	Reverse primer (5'-3')
ADH4	P	Chr.8:121,223,485–121,225,485	Chr.8:121,223,029–121,225,721	2694	ATTTATGGTTGCCAAAGGGAG	GGGAAACTCGCCTTGGAA
	E1	Chr.8:121,037,000–121,039,000	Chr.8:121,037,200–121,038,532	1333	CCTCTGCCACAACCTATCA	AGAAGTAACCGGCTATATCCA
	E2	Chr.8:121,160,000–121,162,000	Chr.8:121,159,670–121,161,891	2222	TTGTTAATTGCCAGTCCCATC	CACTGACCATCAGCCCATAG
PPARα	P	Chr.5:3,300,755–3,302,755	Chr.5:3,300,546–3,302,652	2107	CAGATCCCGTGTTGCTATGGC	GCGGCTTTGCACCTTGGAC
	E1	Chr.5:3,363,000–3,365,000	Chr.5:3,362,956–3,365,047	2092	CACAGCGGTGTAAAGTGACTA	TTGACTTGCACTGCCCTAT
	E2	Chr.5:3,372,000–3,374,000	Chr.5:3,371,911–3,374,044	2134	GGACGGGACGGTGTGTTT	ACAGAGCAAGCGAAGGGTAGG
ADIPOQ	P	Chr.13:124,631,906–124,633,906	Chr.13:124,621,856–124,623,910	2055	CTCAGGGCTTGAAGTTTCCAC	GCACCGCACTGTCTGACT
	E1	Chr.13:124,700,000–124,702,000	Chr.13:124,699,907–124,702,089	2183	CCCTTCGCCTCCACTCCTCTT	CAGCGTGCTTATGCCCAAACA
	E2	Chr.13:124,801,600–124,803,600	Chr.13:124,801,572–124,803,845	2274	TTGTTAAAGGCCTCCGAGCTT	ACAGGGCCCTTCCACTTAGAA
	E3	Chr.13:124,775,000–124,776,000	Chr.13:124,774,704–124,776,146	1443	AAATGCTGAAGCCGTAGACTA	GGCCAGGTTGAAAGAGTGT

	E4	Chr.13:124,810,000–124,811,000	Chr.13:124,809,773–124,810,972	1200	AGTTATGTGGGCAGCATGGAG	GCCCTGGAAGGTCAACTCTC
	E5	Chr.13:124,791,000–124,793,000	Chr.13:124,791,166–124,793,064	1899	ATCACCCTTTATTCTGCCATC	GACCACTCAGGCAACCC
	E6	Chr.13:124,814,000–124,815,000	Chr.13:124,813,912–124,814,950	1039	AAGCCAAGGTAAAGTTGACTG	GATAGCCGACCAAGACAAA
	E7	Chr.13:124,755,000–124,756,000	Chr.13:124,755,079–124,755,693	615	AGGGAGGTGCATGCAAGTAC	TCCCCTACCTCATATCACCCA
	E8	Chr.13:124,767,000–124,769,000	Chr.13:124,767,157–124,768,912	1756	TGACTCTATTCCACTTGCCTT	GCCCATCCAGTGAGTTCATA
	E9	Chr.13:124,805,500–124,806,500	Chr.13:124,805,201–124,806,555	1355	CTTCGTCCACTCGGGTCTC	GCCAAGATCAAACACACCATC
	E10	Chr.13:124,771,100–124,773,100	Chr.13:124,770,939–124,773,491	2553	CACAGAGCCCTGGGTTCAAA	AGGACAATGCCCACACAAAGC
